# Tuning Thermal Stability through Dopant Size in Chemically Doped DPP–Thiophene Polymers

**DOI:** 10.1021/acs.chemmater.5c02923

**Published:** 2026-02-20

**Authors:** Kan Tang, Alyssa Shaw, Yunfei Wang, Yadong Zhang, Rachael J. Warner, Andrew Bates, Naomi Nelson, Chenhui Zhu, Tanguy Terlier, Rafael Verduzco, Derya Baran, Stephen Barlow, Seth R. Marder, Simon Rondeau-Gagné, Xiaodan Gu

**Affiliations:** † Center for Optoelectronic Materials and Devices, School of Polymer Science and Engineering, The University of Southern Mississippi, Hattiesburg, Mississippi 39406, United States; ‡ Renewable and Sustainable Energy Institute, University of Colorado Boulder, Boulder, Colorado 80309, United States; § Department of Chemistry and Biochemistry, 8637 University of Windsor, Windsor, Ontario N9B3P4, Canada; ∥ Advanced Light Source, 1666 Lawrence Berkeley National Laboratory, Berkeley, California 94720, United States; ⊥ SIMS laboratory, Shared Equipment Authority, 3990 Rice University, Houston, Texas 77005, United States; # Department of Chemical & Biomolecular Engineering, 3990 Rice University, Houston, Texas 77005, United States; ∇ Physical Sciences and Engineering Division, KAUST Solar Center, King Abdullah University of Science and Technology, Thuwal 23955, Saudi Arabia; ○ Department of Chemistry, University of Colorado Boulder, Boulder, Colorado 80309, United States; ◆ Department of Chemical and Biological Engineering, University of Colorado Boulder, Boulder, Colorado 80309, United States

## Abstract

Molecular doping of conjugated polymers (CPs) is a key strategy for improving the performance of organic electronics devices, particularly thermoelectrics. Doped donor–acceptor (D–A) conjugated polymers, characterized by a tunable energy gap between the Fermi level and the transport band, show great promise in achieving high electrical conductivity (σ) while preserving a favorable Seebeck coefficient (*S*). Despite the promising performance enhancement of chemically doped D–A polymers, their thermal stability remains largely underexplored, a crucial consideration for the long-term operation of organic thermoelectric devices. In this study, we investigated the dopant size-dependent thermal stability of a diketopyrrolopyrrole-thiophene (DPP-T) D–A copolymer, utilizing two p-dopants: 2,3,5,6-tetrafluoro-7,7,8,8-tetracyanoquinodimethane (F_4_TCNQ) and Mo­(tfd-CO_2_Me)_3_. Temperature-dependent UV–vis–NIR spectroscopy revealed that DPP-T/F_4_TCNQ is more prone to dedoping under a high temperature thermal stress than DPP-T/Mo­(tfd-CO_2_Me)_3_. Although the F_4_TCNQ doped polymer shows higher initial in-plane conductivity than its Mo­(tfd-CO_2_Me)_3_ counterpart, it undergoes a conductivity loss of more than an order of magnitude after annealing at 120 °C for 30 min. In contrast, the in-plane conductivity of DPP-T/Mo­(tfd-CO_2_Me)_3_ remains stable under the same thermal conditions. Thermogravimetric analysis ruled out dopant sublimation as a primary contributor to dedoping, leading us to attribute the conductivity loss in F_4_TCNQ-doped DPP-T to dopant phase separation and migration. This observation was further confirmed by X-ray scattering studies and nanoscale infrared microscopy and spectroscopy studies. This work could provide further insights into the thermal stability of doped conjugated polymers and suggests that incorporating bulkier dopants is an effective strategy to enhance the thermal robustness of doped DPP-type systems.

## Graphical abstract

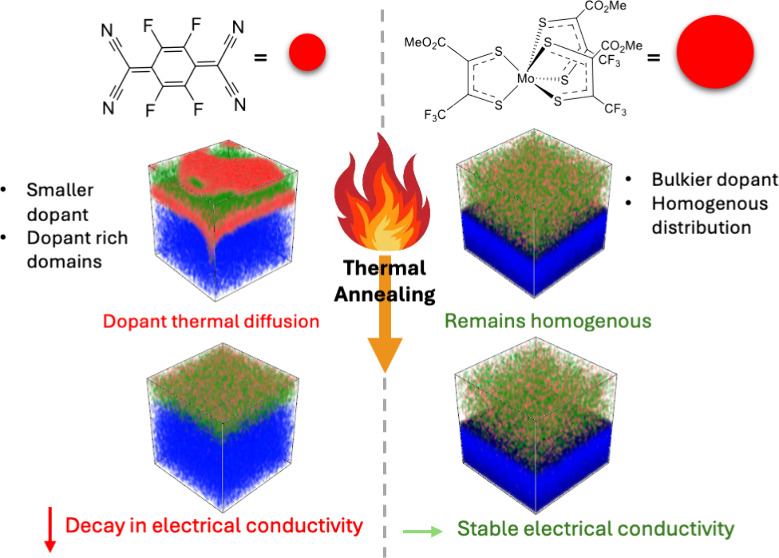

## Introduction

Donor–acceptor conjugated polymers (CPs) have attracted significant attention in organic electronics, particularly in thermoelectric (TE) applications, due to their low thermal conductivity, tunable electrical conductivity, and solution processability.^1–8^ When employed in devices that convert heat into electricity, the performance of a TE material is commonly described by the figure of merit, *ZT* = σ*S* ^2^ *T*/*k*, and the power factor, *PF* = σ*S*,^2^ where σ is electrical conductivity, *S* is the Seebeck coefficient, *T* is temperature, and *k* is thermal conductivity. Thus, optimizing TE performance requires a careful balance of electrical conductivity (σ), Seebeck coefficient (*S*), and thermal conductivity (κ). Although, in principle, *S* and σ are oppositely dependent on the carrier concentration,^3,9,10^ their empirical dependance on σ (*S* ∝ σ^–1/4^ and PF ∝ σ^–1/2^) suggests doping CPs is an effective approach to enhance TE performance, yielding both high *PF* and high *ZT*.^2,3^ Given that CPs inherently possess lower κ values than traditional metallic TE materials such as Bi_2_Te_3_,^3,11^ chemically doped CP systems are promising in achieving competitive TE performance through increased σ and improved PF.^3^ For the CP poly­(3-hexylthiophene) (P3HT), a σ of 10^3^ and a *PF* of 10^2^ μW m^–1^ K^–2^ was achieved upon p-doping with magic blue (MB) by Brinkmann’s group via fine control of the alignment of P3HT and dopant distribution.^12^ Even higher performance (σ ∼ 10^5^ S cm^–1^, *PF* ∼ 1 mW m^–1^ K^–2^) was demonstrated in FeCl_3_-doped poly­(2,5-bis­(3-alkylthiophen-2-yl)­thieno­[3,2-*b*]­thiophene) (PBTTT), employing a similar strategy.^13^ Recently, efforts in TE materials research have increasingly focused on doped donor–acceptor (D–A) conjugated polymers, which offer superior electronic properties due to their extended conjugation and higher persistence lengths than donor-only CPs such as P3HT.^14–17^ Several groups have developed high-performance D–A TE copolymers based on diketopyrrolopyrrole (DPP) acceptor cores,^18–22^ achieving state-of-the-art values of σ ∼ 10^2^ and *PF* ∼ 10^2^ μW m^–1^ K^–2^. More recently, promising n-dopable conjugated polymers, such as poly­(benzodifurandione) (n-PBDF), have also been developed by the Huang and Mei groups.^23,24^

Despite the excellent TE performance achieved with doped CPs, their thermal stability, especially that of D–A polymers, remains a key concern for the long-term operation of TE devices at elevated temperatures. Moulé’s group investigated the thermal stability of 2,3,5,6-tetrafluoro-7,7,8,8-tetracyanoquinodimethane (F_4_TCNQ), a widely used p-dopant, in stratified dopant/organic semiconductor layers such as *N*,*N*,*N*′,*N*′-tetrakis­(4-methoxyphenyl)­benzidine (MeO-TPD)^25^ and P3HT^26,27^ under elevated temperatures ranging from 80 to 200 °C. They observed a loss in conductivity due to dopant diffusion beginning around 80–110 °C, as detected by UV–Vis–NIR spectroscopy, photoluminescence (PL), and neutron reflectometry. Similarly, Hase et al. confirmed F_4_TCNQ drift within the P3HT matrix starting at 60–80 °C using GIWAXS and FTIR, with full dopant desorption observed at 120 °C.^28^ Watts et al. also reported a similar conductivity loss in F_4_TCNQ doped P3HT above 60 °C, attributing it to the conversion of the F_4_TCNQ^•–^ into a weak dopant, HF_4_TCNQ , via hydrogen abstraction or proton transfer from the α-carbon hydrogen on the hexyl chains at the 3-position of the quinoidal thiophene ring of oxidized P3HT, resulting in a closed-shell [P3HT-H]^+^ species with lower hole mobility.^29^ In addition, Watts describes the “segregation” of F_4_TCNQ to the upper portion of doped P3HT films before thermal treatment, after which spectroscopy shows homogenization of the F_4_TCNQ.^29^ However, the focus of their work is not on the dopant size, nor is emphasis placed on the effect of the physical dopant thermal diffusion on electrical properties. In response to the thermal instability of F_4_TCNQ-doped CPs, several mitigation strategies have been proposed. Kroon et al. reported that incorporating oligoethylene glycol (OEG) polar side chains into thiophene monomers enhances the thermal stability of F_4_TCNQ-doped thiophene-based CPs.^30^ The OEG-functionalized polymer p­(g_4_2T-T) maintained stable conductivity upon heating up to 150 °C. Dong et al. found that directly linking the OEG’s oxygen atom to the thiophene ring without a CH_2_ spacer provides greater thermal stability compared to F_4_TCNQ doped unmodified polythiophene with alkyl side chains.^31^ These findings suggest that introducing oligoether side chains strengthens side-chain/dopant interactions, thereby reducing dopant drift and thermal desorption. On the other hand, Watts et al. attributed the improved thermal stability of glycolated P3HT to the suppression of HF_4_TCNQ ^-^ formation, achieved by replacing the labile hydrogen-containing CH_2_ group attached to thiophene with an oxygen atom.^29^ Although significant progress has been made in enhancing the thermal stability of polythiophene/F_4_TCNQ systems through OEG side chains and elucidating various dedoping mechanisms, the thermal behavior of bulkier nonplanar dopants, excluding C_60_F_36_ ^25,32^ and 2,3-di­(*N*-phthalimido)-5,6-dicyano-1,4-benzoquinone (BAPD),^33^ remains largely unstudied for D–A copolymers. This gap represents a key unresolved issue in improving the thermal stability of doped organic TE materials.

In this manuscript, we investigated the dopant size-dependent thermal stability of a doped diketopyrrolopyrrole-terthiophene (DPP-T) D–A copolymer using two p-type dopants, F_4_TCNQ and molybdenum tris­(1-(methoxycarbonyl)-2-(trifluoromethyl)-ethane-1,2-dithiolene) (Mo­(tfd-CO_2_Me)_3_), which have significantly different sizes and molar masses. Despite relatively low electron affinities (EAs) of the dopants (EA_F_4_TCNQ_ ≈ 5.2 eV,^34^ EA_Mo(tfd‑CO2Me)3_ ≈ 5.0 eV^35^), doping of DPP-T was achieved, as evidenced by the emergence of polaron absorption features in UV–vis spectra (1400–1500 nm). UV–vis–NIR spectroscopy showed that dedoping occurs in both F_4_TCNQ-doped and Mo­(tfd-CO_2_Me)_3_-doped DPP-T copolymers starting at 40 °C. However, at 120 °C, over 60% of the polaron signal was retained in Mo­(tfd-CO_2_Me)_3_-doped samples, compared to less than 30% in the F_4_TCNQ doped counterparts. Furthermore, DPP-T doped with Mo­(tfd-CO_2_Me)_3_ retained its in-plane conductivity after annealing at 120 °C for 30 min; whereas, F_4_TCNQ-doped DPP-T experienced a conductivity drop of more than an order of magnitude under the same annealing conditions, demonstrating the superior thermal stability of Mo­(tfd-CO_2_Me)_3_-doped DPP-T. Thermogravimetric analysis (TGA) of both F_4_TCNQ-doped and Mo­(tfd-CO_2_Me)_3_-doped DPP-T copolymers revealed that dopant sublimation only begins well above 120 °C. This indicates that the dedoping and conductivity loss of F_4_TCNQ-doped samples at 120 °C cannot be attributed to dopant sublimation. Instead, the dopant likely undergoes diffusion within the polymer matrix (Figure 1). In contrast, the improved thermal stability of Mo­(tfd-CO_2_Me)_3_ in the same matrix is attributed to its bulkier, three-dimensional molecular structure. We recognize hydrogen abstraction from the polymer’s α-proton, as proposed by Watts et al., may contribute to dedoping, yet it is unlikely, in this case, due to the DPP-T type polymer having the alkyl group on the nitrogen which is off the main chain conjugation path unlike P3HT.^29^ However, the Mo-based dopant shows no evidence of such side reactions. Consistent with Watts’ F_4_TCNQ-doped P3HT study, we confirm that dedoping also arises from thermal dopant diffusion in CPs, which reduces electronic performance, while a bulkier dopant avoids this loss. This work offers new insights into the thermal stability of doped conjugated polymers and suggests that incorporating bulkier dopants is an effective strategy for improving the thermal robustness of doped DPP-type systems.

**Figure 1:**
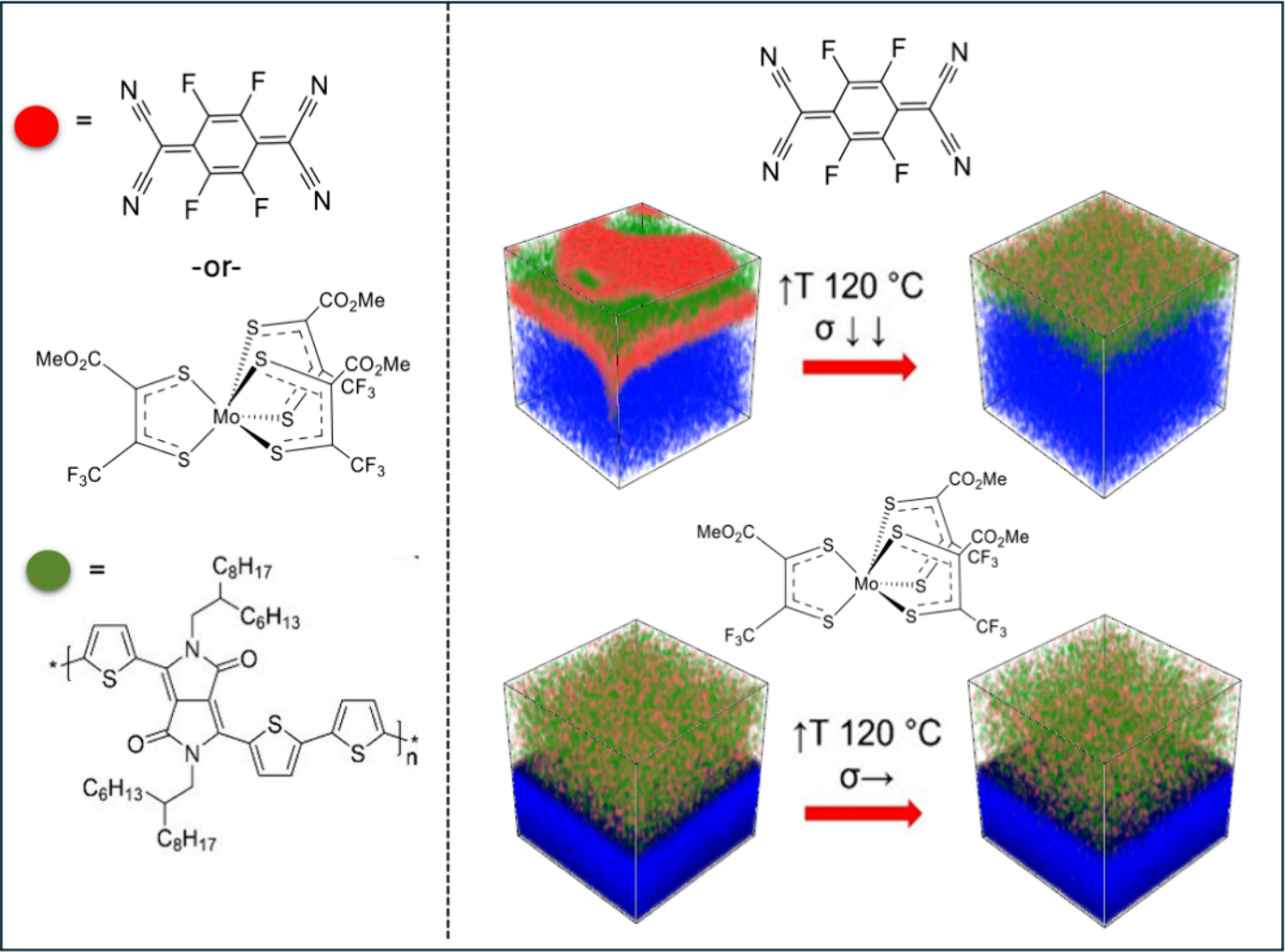
(left) Chemical structures of the DPP-T-C6C8 donor–acceptor copolymer, F_4_TCNQ, and Mo­(tfd-CO_2_Me)_3_. (right) Schematic illustration of the diffusion and redistribution of two dopants in the DPP-T copolymer matrix. Note that the drawings are not drawn to scale.

## Results and Discussion

DPP-T films were obtained by drop casting solutions containing polymer and dopant. In Figure 2a, the UV–vis spectra of doped DPP-T exhibit three prominent absorption peaks at 420, 760, and 1450 nm, respectively. The first two correspond to the neutral polymer, while the last one can be attributed to the doping product, in the form of either charge transfer complex^36^ or DPP-T^+^ polaron.^37,38^ However, the similarity of the doping product peaks at 1400–1500 nm for two different dopants suggests the presence of the polaronic species. Furthermore, doping to form polarons is consistent with reported electrochemical redox potentials for DPP-T polymer (*E*_ox,onset_ = +0.07 V vs. FeCp_2_ ^39^) and the dopants (*E*_1/2, red_ values for F_4_TCNQ and Mo­(tfd-CO_2_Me)_3_ are +0.18 V^40^ and +0.12 V^41^ vs. FeCp_2_, respectively).

**Figure 2:**
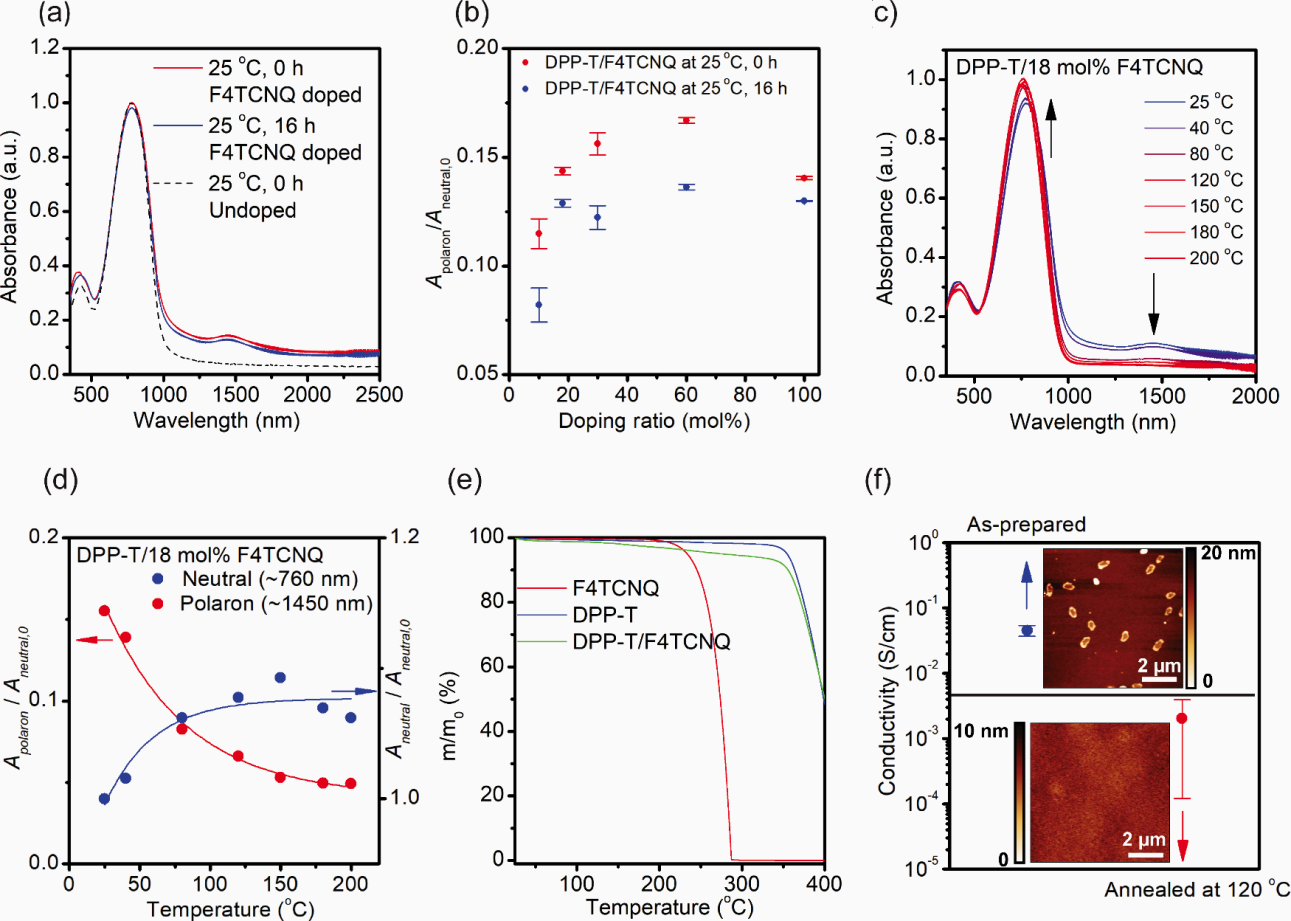
Doping and dedoping behavior of DPP-T with F_4_TCNQ dopants. (a) UV–vis-NIR spectra of drop-cast films of DPP-T doped with 18 mol % F_4_TCNQ before and after being held at ambient temperature (25 °C) for 16 h in air. (b) Estimated relative polaron generation (*A*_polaron_/*A*_neutral, *t*=0_) as a function of doping ratio in air. Doping ratio (mol %) is defined as [dopant]/[DPP-T]_monomer_. (c) UV–vis–NIR spectra of a drop-cast film of DPP-T doped with 18 mol % F_4_TCNQ as a function of annealing temperature in air. Each temperature was maintained for 10 min prior to spectrum acquisition. (d) Evolution of the relative polaron concentration (*A*_polaron_/*A*_neutral, *t*=0_) and normalized neutral polymer peak (*A*_neutral_/*A*_neutral, *t*=0_) as a function of annealing temperature in air. (e) TGA of DPP-T, F_4_TCNQ, and DPP-T doped with 18 mol % F_4_TCNQ, conducted at a heating rate of 1 °C/min in N_2_. (f) In-plane conductivity of a spin-coated DPP-T film doped with 18 mol % F_4_TCNQ before and after thermal annealing at 120 °C for 30 min in N_2_. Inset: AFM topography images (10 × 10 μm^2^) of the as-cast (top) and annealed samples (bottom).

A small but discernible decrease in peak absorbance can be observed in both neutral and polaron features from the UV–vis measurements in air. The decline in polaron absorbance is similarly observed across doping ratios ranging from 10 mol % to 100 mol % (Figure 2b). The slight decrease in the strong neutral peak is similar to that seen in undoped films after 16 h (Figure S1a), possibly due to photobleaching in air. The reduction in polaron absorbance is attributed to dedoping in the DPP-T/F_4_TCNQ film. This observation is consistent with previous reports on slow F_4_TCNQ diffusion in a P3HT film, even at room temperature, which highlights the inherent instability of the doped film even at lower temperatures.^27^ As shown in Figures 2c and 1d, the polaron absorbance decreases significantly above 40 °C for freshly prepared samples, reducing to around 50% of its initial value at 80 °C, and leveling off beyond 150 °C. Concurrently, the absorbance of the neutral peak at 760 nm increases and then stabilizes with rising temperature. This isosbestic transition observed between the neutral DPP-T and polaron peaks during the temperature ramping further confirms temperature-induced dedoping in the DPP-T/F_4_TCNQ film.

To examine the electrical performance of F_4_TCNQ-doped DPP-T films upon thermal stress, in-plane electrical conductivity was measured before and after thermal annealing. As shown in Figure 2f, the in-plane conductivity decreases by more than an order of magnitude, from 4.6 ± 0.9 × 10^–2^ S cm^–1^ to 2.0 ± 1.9 × 10^–3^ S cm^–1^, after annealing at 120 °C for 30 min in N_2_. However, a TGA scan at a heating rate of 1 °C/min suggests that the F_4_TCNQ dopant exhibits no significant mass loss or sublimation at 120 °C, with significant sublimation only observed at temperatures above 200 °C (Figure 2e). To further rule out thermal sublimation as the cause of dedoping, isothermal TGA scans were performed at 120 °C for 24 h on neat dopants and the neat polymer, revealing no mass loss during annealing (Figure S7). An additional isothermal scan at 200 °C for 24 h showed a gradual mass loss, with approximately 70% of the sample mass remaining at the end of the experiment. These results suggest that F_4_TCNQ does not sublime from the doped polymer films at 120 °C. After annealing, the surface roughness of the doped film decreases from 2.6 to 0.7 nm (Figure 2f), accompanied by a reduction in dot-like surface features. Previous AFM-KPFM studies indicated that these dot-like features correspond to the surface precipitation of F_4_TCNQ dopants.^42^

Thus, the thermally induced disappearance of the F_4_TCNQ from the CP film surface upon annealing suggests that the F_4_TCNQ dopant can migrate both laterally and vertically through the film when heated. To investigate the possibility that reactions involving atmospheric water and/or oxygen also contribute to this decay, we also performed the film thermal annealing of doped samples in a N_2_ glovebox, and compared the UV–vis spectra of samples annealed in N_2_ and in air (Figure S14 and S15); the similar loss of the polaronic band in both cases suggests these side reactions do not play a major role in the thermal decay. Given the 90% decline in conductivity at 120 °C (Figure 2f) and the reversion of polarons to the neutral state of DPP-T at the same temperature (Figure 2d), the dedoping is attributed to dopant diffusion and the subsequent displacement of F_4_TCNQ from its doping sites within the D–A polymer matrix. We consider the alternative dedoping mechanism proposed by Watts et al.,^29^ involving thermally induced hydrogen abstraction or proton transfer between F_4_TCNQ^•–^ and the alkyl side chains of the polymer, to be unlikely in this case, where the DPP-T HOMO has a relatively low coefficient on the N atom to which the alkyl group is attached, in contrast to the case of P3HT, where there is a relatively large HOMO coefficient on the 3-carbon, which bears the alkyl chain.^17^ Moreover, we anticipate that this side reaction, while resulting in the loss of polaron absorption, would be unlikely to result in the same spectrum as that of the neutral polymer. However, we cannot completely rule out this mechanism as contributing.

To mitigate dopant diffusion (and perhaps potential side reactions initiated by F_4_TCNQ^•–^) and to study the effect of the dopant size on overall thermal stability, the Mo­(tfd-CO_2_Me)_3_ dopant with bulky ligands was selected for its larger van der Waals volume, which is anticipated to lead to reduced diffusivity within the polymer film, and its different LUMO distribution (delocalized over the C_2_S_2_ portions of the three ligands with no extension onto the substituents in the closely related Mo­(tfd)_3_ ^43^), which will likely lead to a less reactive radical anion. As shown in Figure 3a and 2b, despite the electron transfer between Mo­(tfd-CO_2_Me)_3_ (EA ≈ 5.0 eV) and DPP-T being less exergonic than doping with F_4_TCNQ, a polaron band centered at around 1400 nm is still observed in the UV–vis-NIR spectra of DPP-T doped with Mo­(tfd-CO_2_Me)_3_, confirming successful doping of the DPP-T polymer, albeit with reduced polaron generation, as indicated by a lower polaron to neutral polymer peak ratio (*A*_polaron_/*A*_neutral,0_). Unlike DPP-T/F_4_TCNQ, DPP-T/Mo­(tfd-CO_2_Me)_3_ films at various doping ratios (10–30 mol %) showed no noticeable changes in their polaron peaks at room temperature (Figure S2). This suggests that DPP-T/Mo­(tfd-CO_2_Me)_3_ remains stable at room temperature. Additionally, temperature-dependent UV–vis-NIR spectra indicate improved thermal stability. As shown in Figure 3b, the polaron peak absorbance decreased linearly with temperature, followed by a sharp drop that levels off into a plateau above 150 °C.

**Figure 3:**
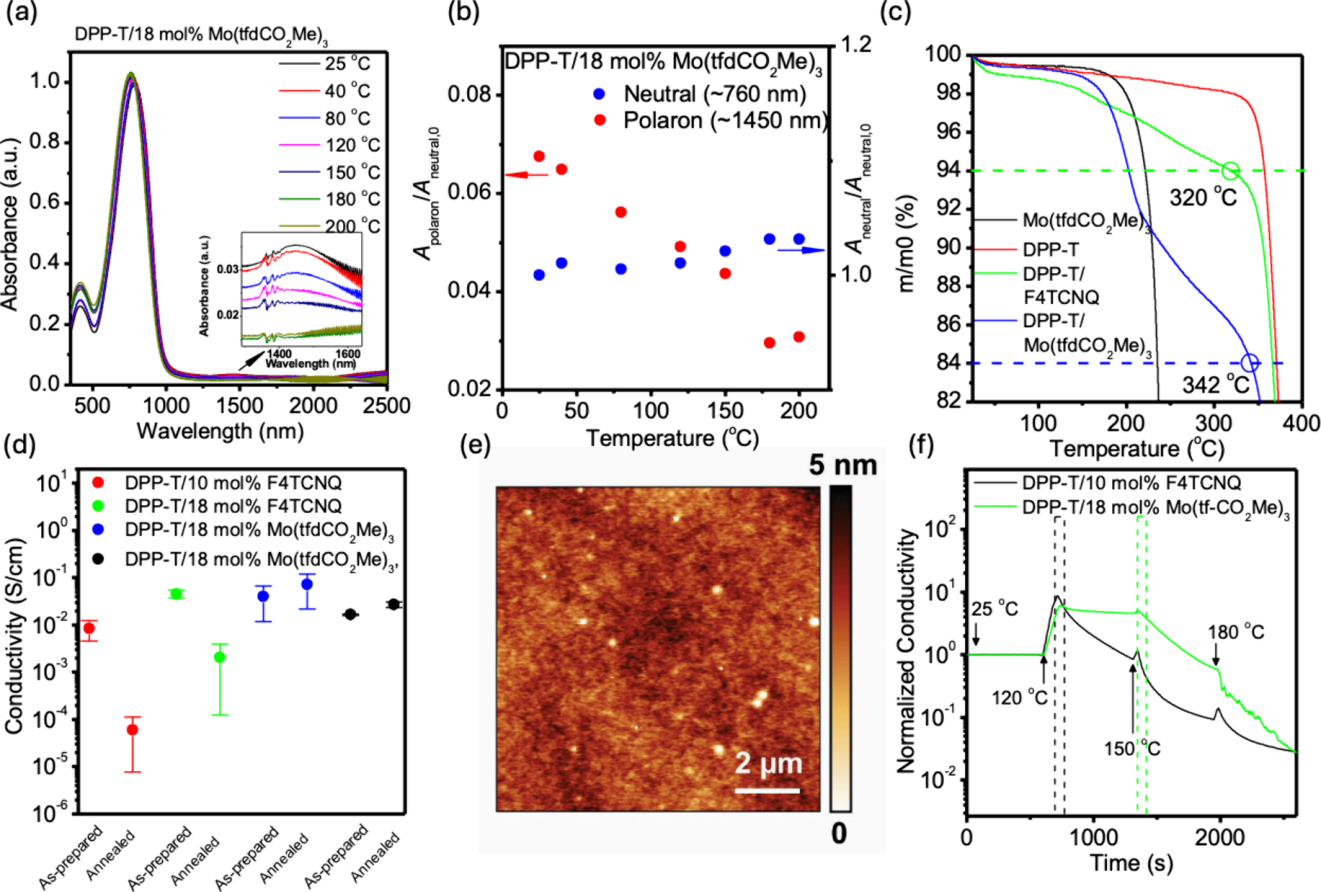
Doping and dedoping behavior of DPP-T with Mo­(tfd-CO_2_Me)_3_ dopants. (a) UV–vis–NIR spectra of drop-cast DPP-T film doped with 18 mol % Mo­(tfd-CO_2_Me)_3_ as a function of temperature in air. Each temperature was maintained for 10 min before acquiring the next spectrum. Inset: expanded view of the 1450 nm region. (b) Evolution of the estimated polaron generation and neutral peak as a function of temperature in air. (c) TGA traces of Mo­(tfd-CO_2_Me)_3_, DPP-T, DPP-T with 18 mol % F_4_TNCQ, and DPP-T with 18 mol % Mo­(tfd-CO_2_Me)_3_ in N_2_, all measured at a heating rate of 1 °C/min. Dashed lines indicate the theoretical total mass losses corresponding to 6 wt % of F_4_TCNQ and 16 wt % of Mo­(tfd-CO_2_Me)_3_, which are equivalent to 18 mol % loading of each dopant. (d) Summary of in-plane conductivity measurement of doped DPP-T film with varying thicknesses before and after thermal annealing at 120 °C for 30 min in N_2_. (e) AFM topography image (10 × 10 μm^2^) of a DPP-T film with 18 mol % Mo­(tfd-CO_2_Me)_3_. (f) Normalized *in situ* electrical conductivity measurements of doped DPP-T films during a programmed temperature ramp from 25 to 200 °C in N_2_ (both films were 65 nm thick). Each temperature was held for 10 min during the heating cycle. The dashed areas indicate the onset temperatures where conductivity decay is observed.

Notably, improved doping stability is observed in DPP-T with Mo­(tfd-CO_2_Me)_3_ upon thermal annealing at 120 °C. In Figure 3d, the in-plane conductivities of DPP-T films doped with 18 mol % Mo­(tfd-CO_2_Me)_3_ remain unchanged at 10^–2^ S cm^–1^ after annealing at 120 °C, regardless of film thickness (65 or 140 nm). On the contrary, DPP-T with F_4_TCNQ films exhibited a conductivity drop of more than 90% under the same thermal annealing conditions. To further examine thermal stability, real-time in-plane conductivity measurements were performed during continuous thermal annealing, rather than only before and after heating (Figure 3f). As observed, DPP-T films doped with F_4_TCNQ showed nearly a one-order of magnitude drop in conductivity during the temperature ramp from 120 to 150 °C, whereas Mo­(tfd-CO_2_Me)_3_ doped films maintain stable conductivity below 150 °C. However, once the annealing temperature exceeds 150 °C, a noticeable decline in conductivity is also observed in Mo­(tfd-CO_2_Me)_3_ doped films. Notably, Mo­(tfd-CO_2_Me)_3_-doped films retain a smooth, featureless surface morphology with consistent RMS roughness before and after thermal annealing (Figure 3e and Figure S4), indicating minimal phase separation of the dopant on the film surface following spin-coating and annealing. This behavior is attributed to the improved solubility and stronger polymer/dopant interaction of Mo­(tfd-CO_2_Me)_3_ with DPP-T.

The marked difference in thermal stability between Mo­(tfd-CO_2_Me)_3_- and F_4_TCNQ-doped PDPP-T can be ascribed to the difference in their molecular size and molar mass, as well as improved miscibility of Mo­(tfd-CO_2_Me)_3_ with the polymer matrix, enabled by the incorporation of the methyl ester groups into the molybdenum complex.

Next, we attempted to utilize contact angle analysis to calculate surface energy as an estimation for the χ-interaction parameter between the dopants and polymer. The F_4_TCNQ was unable to form a continuous film on the hydrophilic plasma etched wafers, and we ascribe the hydrophilic contact angle result for the F_4_TCNQ sample (37°) to interference from the plasma etched substrate (26°). The DPP-T polymer as well as the Mo­(tfd-CO_2_Me)_3_ dopant formed uniform films with average contact angles of 90° and 97°, respectively (Supporting Figure S11). The contact angle analysis results are summarized in the Supporting Information. We recognize that the miscibility difference between the dopants could influence the initial doped film morphology and it is likely this is represented in the phase separated F_4_TCNQ-doped polymer films.

As previously discussed, the drastic (>90%) decrease in conductivity of F_4_TCNQ doped films is not accompanied by an equivalent degree of sublimation loss. Therefore, dopant diffusion within the DPP-T matrix, along with potential side reactions between radical anions and side chains, primarily contributed to the dedoping process. Due to its larger size and 3D geometry, Mo­(tfd-CO_2_Me)_3_ diffuses more slowly through the DPP-T matrix than F_4_TCNQ. Indeed, minimal decay in conductivity is observed before reaching 150 °C, indicating that the thermal diffusion of Mo­(tfd-CO_2_Me)_3_ is largely impeded below this temperature. To further test this hypothesis, Grazing Incidence Wide-Angle X-ray Scattering (GIWAXS), as well as atomic force microscopy coupled with infrared spectroscopy (AFM-IR), were conducted on neat undoped polymer, as-cast doped samples, and doped samples annealed at 120 °C, mimicking those used in conductivity measurements, to observe the effects of both dopants on the ordering and packing of the DPP-T polymer (Figure 4 and Table 1). In the F_4_TCNQ-doped film, a slight decrease of 0.02 A^–1^ in the (100) alkyl chain stacking peak was observed (Table 1), consistent with the intercalation of F_4_TCNQ in the lamellar region of the polymer.^12,44,45^ In contrast, the lamellar spacing in the Mo­(tfd-CO_2_Me)_3_-doped DPP remained largely unchanged, suggesting the Mo dopant primarily resides in the amorphous region of doped DPP-T. The (010) peak in the F_4_TCNQ-doped sample was not assigned due to potential overlap with sharp dopant peaks­(Table 1). Notably, the samples exhibit no clear or distinct (010) peak, and thus no peak assignment was made.

**Figure 4:**
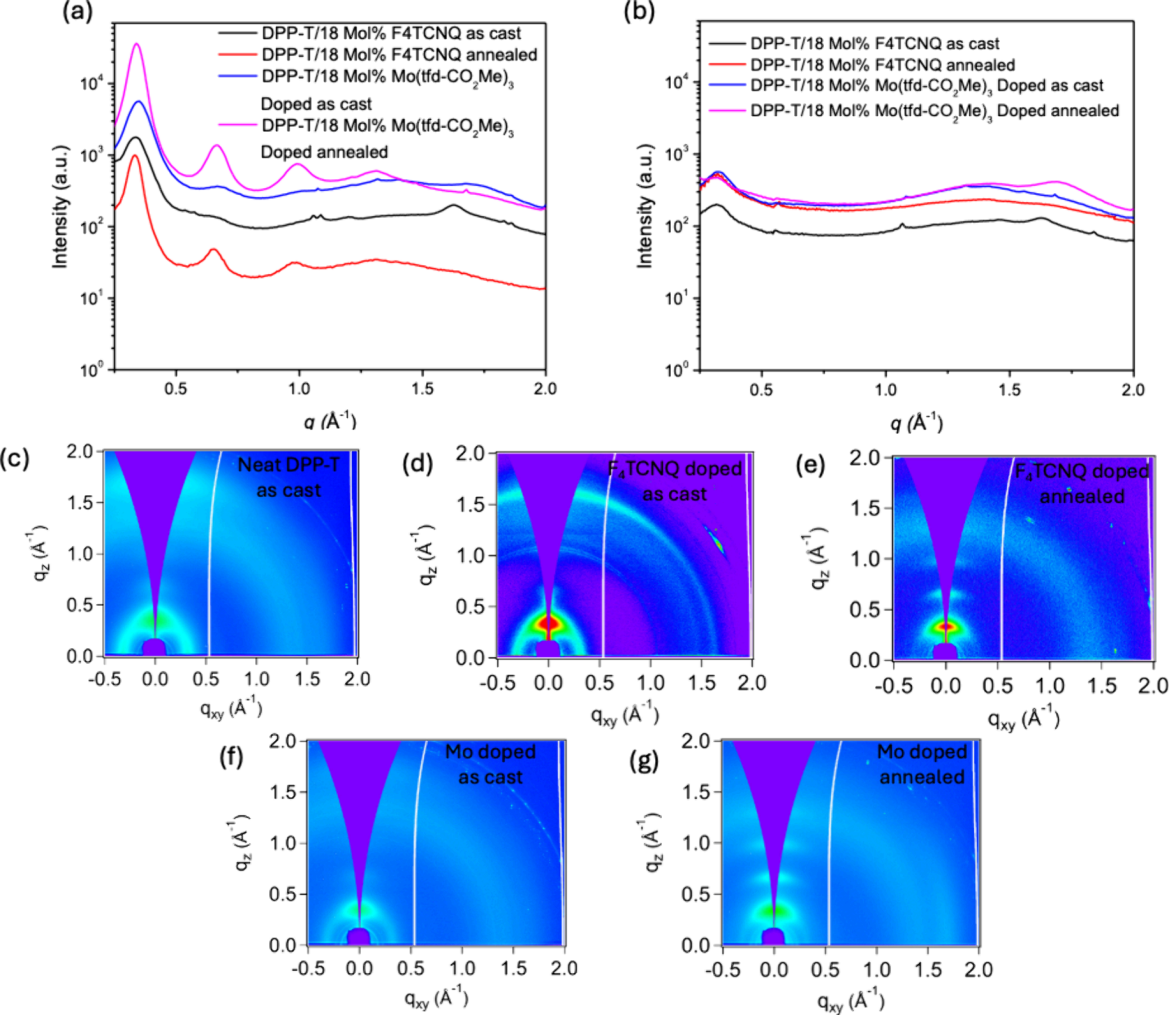
Out-of-plane (a) and in-plane (b) 1D GIWAXS line cuts of doped DPP-T spin-coated films before and after annealing. (c–g) 2D GIWAXS patterns of doped DPP-T spin coated films before and after annealing. (c) Neat DPP-T as cast. (d) As-cast DPP-T with 18 mol % F_4_TCNQ. (e) Annealed DPP-T with 18 mol % F_4_TCNQ. (f) As-cast DPP-T with 18 mol % Mo­(tfd-CO_2_Me)_3_. (g) Annealed DPP-T with 18 mol % Mo­(tfd-CO_2_Me)_3_. The thermally annealed samples were annealed then cooled at ambient temperature.

**Table 1:** Summary of the Peak Positions from the GIWAXS Analysis of the Doped DPP-T Spin-coated Films Before (as-cast), after Thermal Annealing, and Neat DPP-T films

peak assignment	neat DPP-T *q* (Å^–1^)	F_4_TCNQ as-cast *q* (Å^–1^)	F_4_TCNQ annealed *q* (Å^–1^)	Mo(tfd-CO_2_Me)_3_ as-cast *q* (Å^–1^)	Mo(tfd-CO_2_Me)_3_ annealed *q* (Å^–1^)
(100)	0.35	0.33	0.33	0.35	0.34
(200)	0.65	N/A	0.65	0.65	0.66
(300)	N/A	N/A	0.99	N/A	1.00
(010)	1.68	N/A	N/A	N/A	N/A
*d*_alkyl_	17.94	19.03	19.03	17.94	18.47
*d*_π atacking_	3.74	N/A	N/A	N/A	N/A

GIWAXS analysis was also conducted on the doped samples after thermal annealing at 120 °C for 30 min. As shown in Figure 4a, the (100) peaks in both F_4_TCNQ and Mo­(tfd-CO_2_Me)_3_ doped samples became significantly sharper after thermal annealing, and are accompanied by the emergence of (200) and (300) reflections. Notably, the sharp peak observed in the GIWAXS of the F_4_TCNQ-doped DPP-T (*q* of ∼ 1.63 A^–1^) disappeared after thermal annealing (Figure 4b). No significant (010) peak is detected in the Mo­(tfd-CO_2_Me)_3_-doped sample, consistent with the unannealed Mo­(tfd-CO_2_Me)_3_-doped sample. The sharpening of the (*h*00) peaks following annealing is indicative of enhanced lamellar packing order in both doped samples (Figure 3g). However, the disappearance of the sharp peak in the F_4_TCNQ-doped DPP-T upon annealing indicated disruption of the π–π stacking of F_4_TCNQ dopants in the polymer matrix, suggesting thermal diffusion and subsequent redistribution of the F_4_TCNQ dopant. In contrast, minimal changes in the lamellar domain of the Mo­(tfd-CO_2_Me)_3_ doped DPP-T after annealing show limited dopant relocation, consistent with its superior thermal stability at 120 °C compared to F_4_TCNQ.

Next, AFM-IR was also performed to probe the thermally induced redistribution of dopants. Previously, AFM-IR has proven to be a powerful technique to selectively identify the spatial distribution of specific phases within complex polymer composites. For instance, our group has utilized AFM-IR to reveal the spatial location and composition of DPP-based conjugated polymers in both binary phase and ternary systems^6,46–49^ First, bulk FTIR measurements of the DPP polymer, F_4_TCNQ, and Mo­(tfd-CO_2_Me)_3_ were performed to identify characteristic absorption peaks that could be used to track each component in the polymer/dopant system (Figure S12). Additionally, to confirm the peak locations and proper tuning of our AFM-IR scans, traditional ATR-FTIR scans on the solid powders of the DPP-T and each dopant were performed (Figure S13). Combining the spectra from bulk FTIR of AFM-IR and powder ATR-FTIR, a laser wavelength of 1596 cm^–1^ (dopant resonance band) was selected to probe the F_4_TCNQ, while 1750 cm^–1^ (carbonyl group) was used to detect the Mo­(tfd-CO_2_Me)_3_, and 1666 cm^–1^ (C=C double bond in DPP core) was used to detect the DPP-T polymer.^8^ In the F_4_TCNQ-doped sample, the AFM-IR signals at 1596 cm^–1^ were relatively uniform prior to annealing (Figure 5b), suggesting no significant phase segregation between F_4_TCNQ and DPP-T matrix in the as-cast film. After thermal annealing, increased heterogeneity was observed (Figure 5e), with localized ring-like regions exhibiting elevated signal intensity. Furthermore, another characteristic F_4_TCNQ IR signal at 1470 cm^–1^ became more pronounced after thermal annealing, as indicated by the arrows in Figure 5f. These findings suggest that thermal annealing led to a redistribution of F_4_TCNQ, resulting in dopant heterogeneity and surface/vertical segregation. On the other hand, the Mo­(tfd-CO_2_Me)_3_-doped samples exhibited little to no change in morphology or dopant distribution (Figure 6). The AFM-IR image acquired with 1750 cm^–1^ excitation showed a similarly uniform dopant distribution in the as-cast sample. (Figure 5b) More interestingly, after thermal annealing, no apparent phase separation between DPP and Mo­(tfd-CO_2_Me)_3_ was observed as shown in Figure 5e. IR nanospectroscopy data collected from multiple locations on the sample surface showed strong consistency (Figure 5c,f), indicating no phase separation between the polymer donor and Mo­(tfd-CO_2_Me)_3_ dopant. This observation aligns with the spectroscopic and electrical characterization results of the Mo-doped sample at 120 °C, supporting its superior thermal stability.

**Figure 5:**
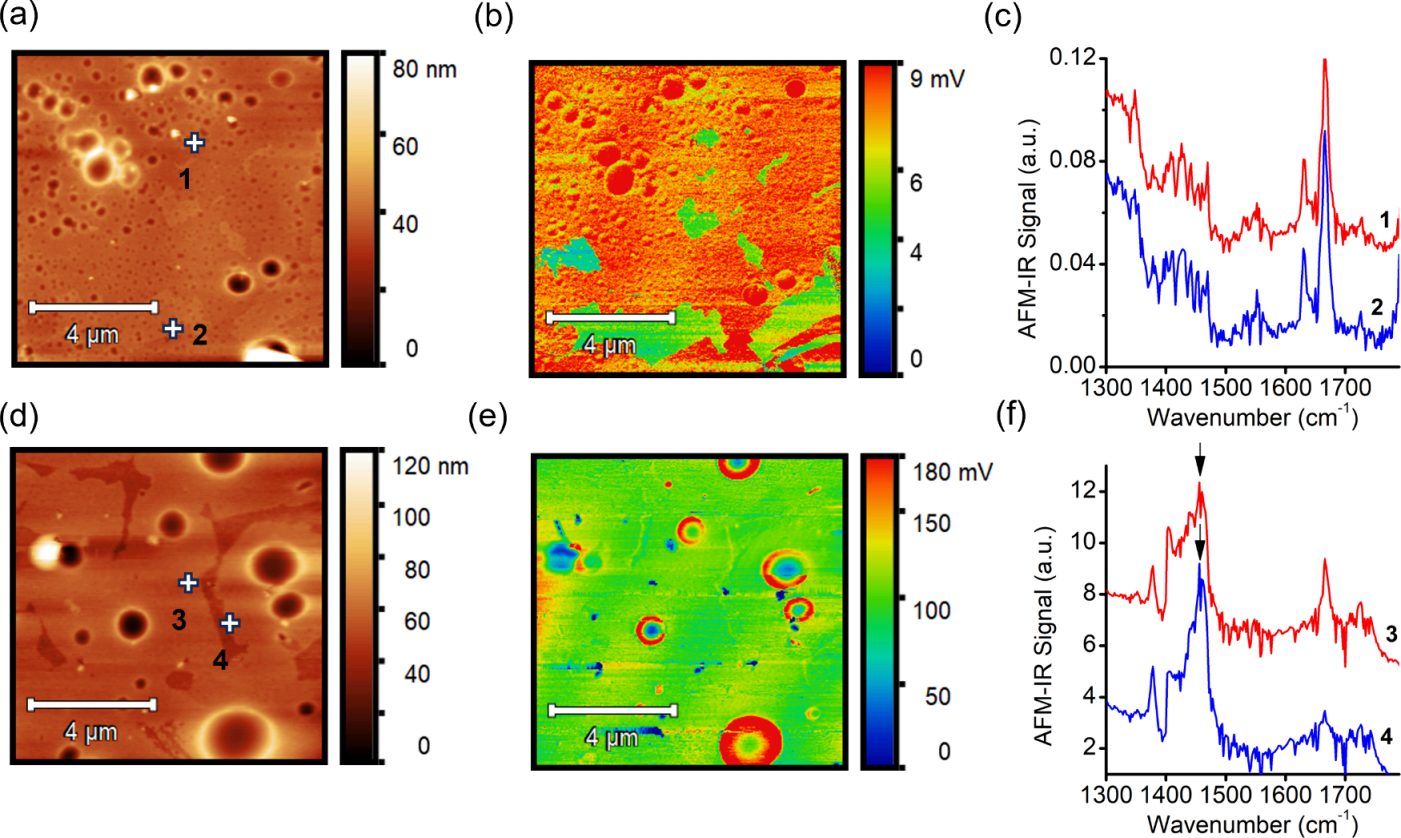
AFM-IR characterization of the as-cast and annealed F_4_TCNQ-doped films. (a) AFM topography, (b) corresponding AFM-IR signal at 1596 cm^–1^ to excite the F_4_TCNQ phase, and (c) nano IR spectra at two representative locations. (d–f) AFM topography, AFM-IR signal at 1596 cm^–1^, and representative IR spectra of the thermally annealed film, respectively. Note that all images are 10 × 10 μm^2^. The AFM-IR signal at 1596 cm^–1^ is a characteristic absorption feature of the F_4_TCNQ dopant.

**Figure 6:**
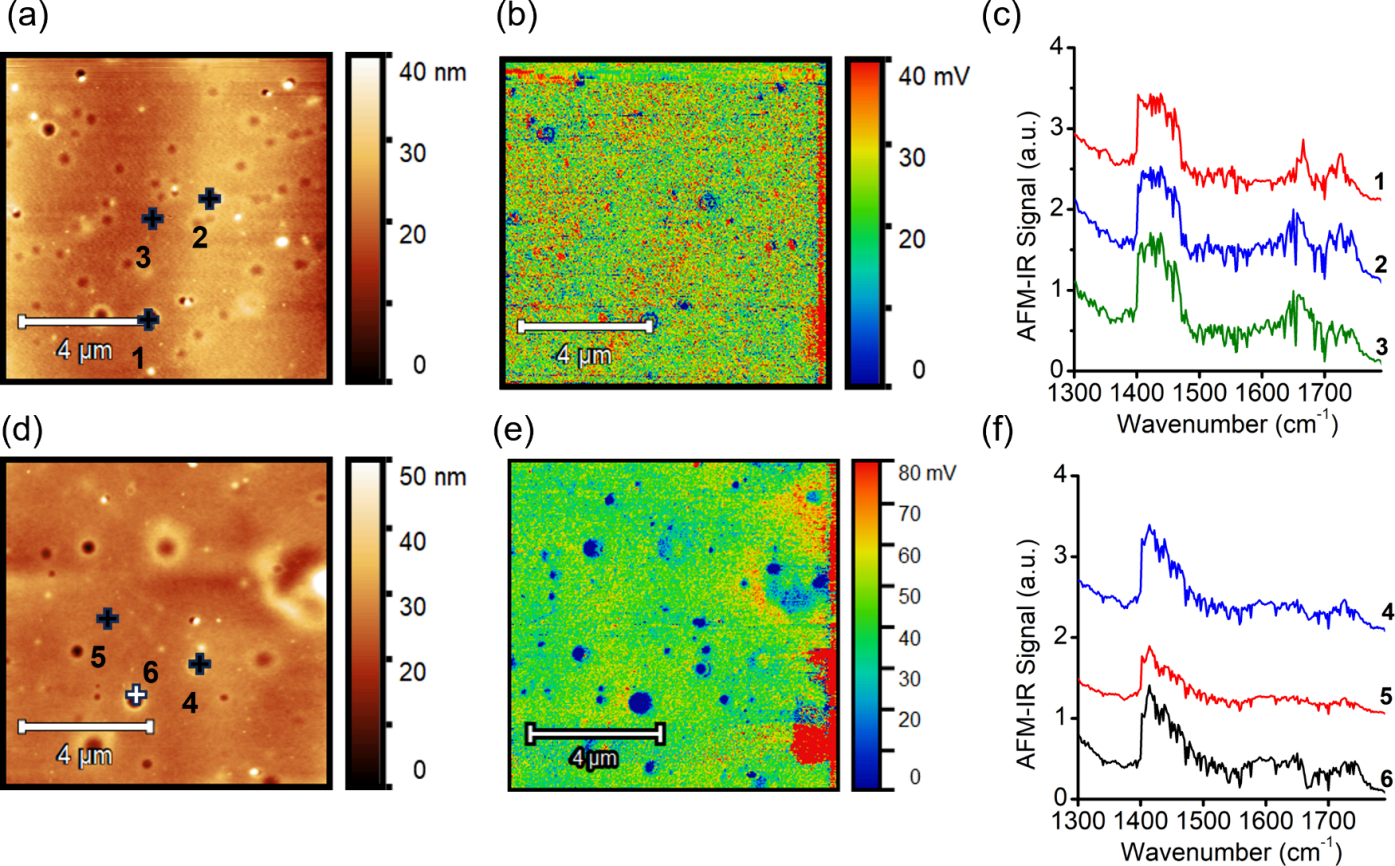
AFM-IR characterization of the as-cast and annealed Mo­(tfd-CO_2_Me)_3_-doped films. (a) AFM topography, (b) corresponding AFM-IR signal at 1750 cm^–1^ to excite the Mo­(tfd-CO_2_Me)_3_ phase, and (c) nano IR spectra at three representative locations. (d–f) show the AFM topography, AFM-IR signal at 1750 cm^–1^, and representative IR spectra of the thermally annealed film, respectively. Note that all images are 10 × 10 μm^2^. The AFM-IR signal at 1750 cm^–1^ is a characteristic absorption feature of the Mo­(tfd-CO_2_Me)_3_ dopant.

Although the AFM-IR analysis provided evidence of dopant segregation before annealing and subsequent dopant migration after annealing, this technique was not able to determine if the dopant diffusion also occurs throughout the film depth. Additionally, the characteristic C≡N bond stretching modes of F_4_TCNQ were not visible in the AFM-IR spectra. We sought to further confirm the dopant thermal diffusion behavior by conducting Time-of-Flight Secondary Ion Mass Spectrometry (TOF-SIMS) in which we could obtain a spectroscopic depth profile of the doped polymer films before and after annealing at 120 °C, mimicking the condition of conductivity experiments. TOF-SIMS analysis revealed that the F_4_TCNQ-doped films show phase separation of the dopant in the films laterally and horizontally (Figure 7a). Depth profile spectroscopy shows surface aggregation of the dopant in the film as well as F_4_TCNQ-rich domains within the film depth (Figure S16 and S17). Notably, after annealing, the F_4_TCNQ surface features disappear (Figure 7b), and the F_4_TCNQ redistributes throughout the depth of the film (Figure S18 and S19). This result agreed well with the AFM measurements, where surface aggregates of F_4_TCNQ can be observed before annealing, yet disappear after annealing. On the contrary, the Mo­(tfd-CO_2_Me)_3_-doped films showed no such dopant segregation (Figure 7c), with the Mo spectra showing uniform distribution throughout the film depth and surface before and after annealing (Figure S20 to S23). This further confirmed the previous experimental evidence that the Mo­(tfd-CO_2_Me)_3_ dopant is spatially more stable against thermal stress than the smaller F_4_TCNQ dopant.

**Figure 7:**
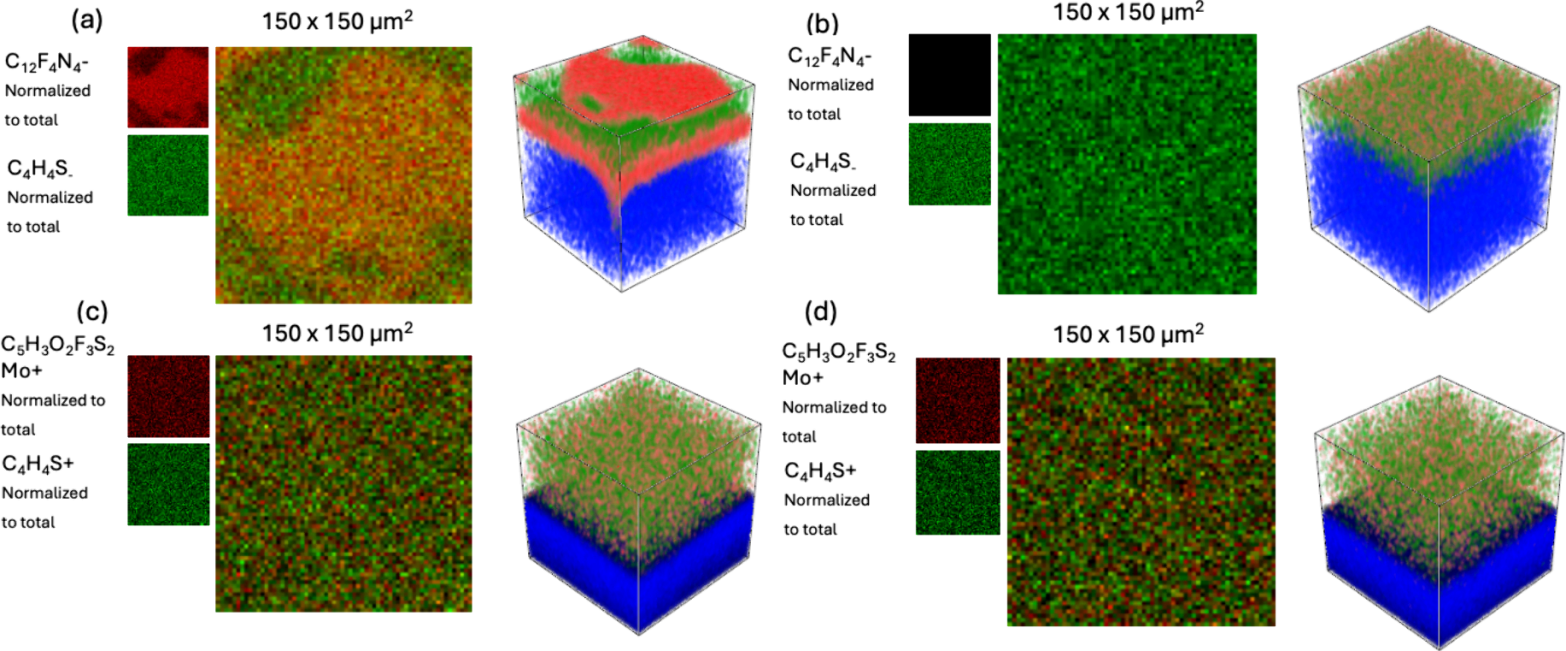
(a) C_12_F_4_N- (red)/C_4_H_4_S- (green) normalized total surface scan (right) and 3D depth profile (left) of an as-cast F_4_TCNQ-doped film, (b) C_12_F_4_N- (red)/C_4_H_4_S- (green) normalized total surface scan (right) and 3D depth profile (left) of an annealed F_4_TCNQ-doped film, (c) C_5_H_3_O_2_F_3_S_2_Mo^+^ (red)/C_4_H_4_S^+^ (green) normalized total surface scan (right) and 3D depth profile (left) of as cast Mo­(tfd-CO_2_Me)_3_ doped film, (d) C_5_H_3_O_2_F_3_S_2_Mo^+^ (red)/C_4_H_4_S^+^ (green) normalized total surface scan (right) and 3D depth profile (left) of an annealed Mo­(tfd-CO_2_Me)_3_-doped film. All data were normalized to the total intensity.

## Conclusions

Overall, DPP-T doped with Mo­(tfd-CO_2_Me)_3_ exhibits enhanced thermal stability compared to its F_4_TCNQ doped counterpart, as evidenced by UV–vis–NIR spectroscopy and conductivity measurements. The temperature-dependent UV–vis spectra shows a slower decay of the polaron signal in the Mo­(tfd-CO_2_Me)_3_-doped DPP-T at 120 °C. Furthermore, its electrical conductivity remains stable at 10^–2^ S cm^–1^ after annealing at 120 °C;whereas, the F_4_TCNQ-doped film loses over 90% of its initial conductivity under the same conditions. Thermogravimetric analysis confirms that dopant sublimation is not the primary cause of dedoping at 120 °C, as both dopants exhibit sublimation temperatures above 200 °C and show no measurable mass loss during isothermal heating at 120 °C for up to 24 h. GIWAXS analysis reveals changes in lamellar and π–π stacking distances in the F_4_TCNQ-doped films, with the disappearance of the sharp (010) peak after annealing indicating dopant diffusion. In contrast, the Mo­(tfd-CO_2_Me)_3_-doped samples showed little to no structural disruption, suggesting limited diffusion due to the dopant’s bulkier structure. AFM-IR and TOF-SIMS measurements further confirmed the phase separation and vertical migration of F_4_TCNQ within the polymer matrix, while the Mo­(tfd-CO_2_Me)_3_-doped sample showed no such phase segregation. Together, these findings highlight the potential of using bulkier dopants to improve the thermal stability of doped donor–acceptor conjugated polymers, thereby advancing the performance and reliability of organic electronic devices such as thermoelectric systems.

## Experimental Section

### Materials

The DPP-C_6_C_8_-T polymer (*M*_w_ = 125–153 kDa) was synthesized by a previously reported method.^15^ F_4_TCNQ (>98%) dopant was purchased from TCI America and used without further purification. Mo­(tfd-CO_2_Me)_3_ dopant was synthesized by following a previous report.^35^ Anhydrous chlorobenzene used for the dissolution of polymers and dopants was from Sigma-Aldrich. Glass slides (2 cm × 2 cm × 0.17 mm) were purchased from Fisher Scientific. Both Si substrates with 300 nm SiO_2_ thermal oxide layer and Si substrates were obtained from University Wafer.

### Preparation of Doped DPP-T Films

For drop-cast films that were used in UV–vis–NIR, 1 mg mL^–1^ dissolved DPP-T/chlorobenzene (CB) solution heated at 80 °C was mixed with an appropriate amount of dissolved F_4_TCNQ/CB (0.2 mg mL^–1^) or Mo­(tfd-CO_2_Me)_3_/CB (1 mg mL^–1^) to achieve desired doping ratio, such as 10 mol % or 18 mol %. Then the mixture was heated at 80 °C for 15 min to ensure complete mixing. Some of the doped mixtures were heated overnight at 80 °C to verify whether additional heating promotes higher polaron generation (Figure S1 and S2). Afterward, the mixture was dispensed onto a glass slide and left to dry in a covered Petri dish, resulting in a solid doped film after drying. For the thermogravimetric analysis samples, a similar procedure to UV–vis–NIR sample preparation was followed, except a higher DPP-T concentration (5 mg mL^–1^) was used to meet the mass requirement (>2 mg) of the instrument. Also, the dried drop-casted films for TGA were subjected to vacuum at 10^–3^ bar for 15 min before being scraped off from the silicon substrate with a sharp blade.

### Characterization of Doped DPP-T Films

UV–vis–NIR absorption spectra were recorded on an Agilent Cary 5000 UV–vis–NIR spectrometer. A Linkam temperature controller was used to control the temperature of the sample holder in a stepwise temperature ramping experiment. The heating rate was set at 50 °C/min, and each temperature was maintained for 10 min.

The thicknesses of the DPP-T films were determined by a Bruker DektakXT stylus profilometer via scanning over sharp scratches made on the SiO_2_/Si substrate. The surface morphology of the DPP-T films on SiO_2_/Si or Si substrate was obtained by Atomic Force Microscopy (Asylum Research) in semicontact mode. The AFM images were processed by Gwyddion software (http://gwyddion.net/, Czech Metrology Institute).

TGA was performed in a Mettler Toledo TGA instrument by monitoring the mass evolution from room temperature (25 °C) to elevated temperatures at a heating rate of 1 °C/min in an inert nitrogen environment.

### Electrical Measurements

SiO_2_ (300 nm)/Si substrates were cleaned by oxygen plasma for 20 min before transferring into a thermal evaporation deposition chamber. Gold electrodes (60 nm) with a 2 nm Chromium adhesion layer were thermally deposited on SiO_2_ (300 nm)/Si substrates at 10^–6^ Torr by using a patterned shadow mask, resulting in a well-defined channel length (*L*) of 30–80 μm and width (*W*) of 1 mm. A solution of DPP-T polymer in chlorobenzene (CB) solution (15 mg mL^–1^) was mixed with either F_4_TCNQ/CB or Mo­(tfd-CO_2_Me)_3_/CB to achieve the desired doping ratio. Doped DPP-T films were spin-coated onto the Au-deposited substrate. The details of spin–coating parameters and resultant film thicknesses can be found in Table S1. The devices were tested on a Signatone 1160 series probe station coupled to a Keithley 4200 semiconductor characterization system inside a nitrogen-filled glovebox. The conductivities (σ) of the doped DPP-T films were calculated from the following expression,

$$\sigma =G\frac{L}{Wd}$$

where *G* is the conductance, *L* is the channel length, *W* is the channel width, and *d* is the thickness of the DPP-T film, respectively. The conductance (*G*) of each film was extracted from the slope of its measured *I*–*V* curve. The reported conductivity was averaged from at least 5 different devices.

### Grazing Incidence Wide Angle X-ray Scattering

The GIWAXS measurements were acquired using beamline 7.3.3 at the Advanced Light Source in the Berkeley Lawrence National Laboratory. The data was acquired under a helium environment with an X-ray beam energy of 10 keV and an incident angle of 0.14°. The samples were thin films spin-coated on plasma-etched silicon wafers. The scattering signal was collected via a Pilatus 2 M detector, and the results were analyzed using the Igor 9 software kit as well as the Irena and Nika package along with WAXSTools. The sample-to-detector distance was 277 mm.

### Atomic Force Microscopy with Infrared Spectroscopy (AFM-IR)

AFM-IR was performed using nanoIR3 from Bruker Instrument, coupled to a MIRcat-QT quantum cascade mid-infrared laser. The AFM-IR data, both images and nanospectroscopy, was collected in tapping mode using a gold-coated AFM probe. The pulse mid-IR laser was tuned to frequencies unique to each component as determined by FTIR characterization.

### Time-of-Flight Secondary Ion Mass Spectrometry

Positive high mass resolution depth profiles were performed using a TOF-SIMS NCS instrument, which combines a TOF.SIMS 5 instrument (ION-TOF GmbH, Münster, Germany) and an in situ Scanning Probe Microscope (NanoScan, Switzerland) at Shared Equipment Authority from Rice University. The analysis field of view was 150 × 150 μm^2^ (Bi_3_^+^ @ 30 keV, 0.3 pA) with a raster of 128 by 128 along the depth profile. A charge compensation with an electron flood gun has been applied during the analysis. An adjustment of the charge effects has been operated using a surface potential. The cycle times were fixed to 200 μs (corresponding to *m*/*z* = 0 – 911 a.m.u mass range). The sputtering raster was 500 × 500 μm^2^ (Ar_1500_^+^ @ 10 keV, 0.2 nA). The beams were operated in noninterlaced mode, alternating one analysis cycle and one sputtering cycle (corresponding 1.63 s) followed by a pause of 5 s for charge compensation with an electron flood gun. All depth profiles have been point-to-point normalized by the total ion intensity and the data have been plotted using a 3-points adjacent averaging. Both normalization and smoothing have permitted a better comparison of the data from the different samples.

## Supplementary Material



## Notes

Additional UV–vis spectrum of the sample, additional AFM images, *I*–*V* measurement of the sample, additional GIWAXS, TGA, FTIR, contact angle, and TOF-SIMS (PDF)
