# Supplementary material for: Tuning Thermal Stability through Dopant Size in Chemically Doped DPP–Thiophene Polymers
Source: Chem Mater. 2026 Feb 20;38(5):2293–304. doi: 10.1021/acs.chemmater.5c02923 (PMC12980630; doi:10.1021/acs.chemmater.5c02923)
Supplement: Supplementary file 1 [file cm5c02923_si_001.pdf]

Supporting information

Tuning Thermal Stability through Dopant Size in Chemically Doped DPP–Thiophene Polymers

Kan Tang[1§], *Alyssa Shaw*[1§], *Yunfei Wang*[1], *Yadong Zhang*[2], *Rachael J. Warner*[3], *Andrew Bates*[1], *Naomi Nelson*[1], *Chenhui Zhu*[4], *Tanguy Terlier*[5,6], *Rafael Verduzco*[6], *Derya Baran*[7], *Stephen Barlow*[2], *Seth R. Marder*[2,9], *Simon Rondeau-Gagné*[3], *Xiaodan Gu*[1*]

[1]Center for Optoelectronic Materials and Devices, School of Polymer Science and Engineering, The University of Southern Mississippi, Hattiesburg, Mississippi 39406, United States

[2]Renewable and Sustainable Energy Institute, University of Colorado Boulder, Boulder, Colorado 80309, United States

[3]Department of Chemistry and Biochemistry, University of Windsor, Windsor, ON N9B3P4, Canada

[4]Advanced Light Source, Lawrence Berkeley National Laboratory, Berkeley, California 94720, United States

[5] SIMS laboratory, Shared Equipment Authority, Rice University, Houston, TX 77005, United States

[6] Department of Chemical & Biomolecular Engineering, Rice University, Houston, TX 77005, United States

[7] Physical Sciences and Engineering Division, KAUST Solar Center, King Abdullah University of Science and Technology, Thuwal 23955, Saudi Arabia

[8] Department of Chemistry, University of Colorado Boulder, Boulder, Colorado 80309, United States

[9] Department of Chemical and Biological Engineering, University of Colorado Boulder, Boulder, Colorado 80309, United States

[*]Email: Xiaodan.gu@usm.edu

[§]K.T. and A.S. contributed equally to this work.

Contents

1. Additional UV-vis spectra of drop-casted DPP-T/F4TCNQ films. 3
2. Processing parameters of spin-coated doped DPP-T-C_6C_8 films 5
3. Additional AFM images. 6
4. *I-V* curves of the doped DPP-T films under room temperature and after thermal annealing......8
5. Supplementary GIWAXS Results 9
6. Supplementary Thermogravimetric analysis (TGA) Results 10
7. Supplementary water contact angle analysis results of thin film DPP-T, F4TCNQ, and Mo(tfd-$CO_2Me)_3$ 12
8. Supplementary FTIR spectra of bulk DPP-T, F4TCNQ, and Mo(tfd-$CO_2Me)_3$ from AFM-IR 12
9. ATR-FTIR Spectra of DPP-T Polymer and Dopants 13
10. UV-Vis study with sample preparation and heating performed in an inert glovebox............14
11. Supplementary TOF-SIMS Point to Point normalization chemical depth profiling 16

1. Additional UV-vis spectra of drop-casted DPP-T/F_4TCNQ films.

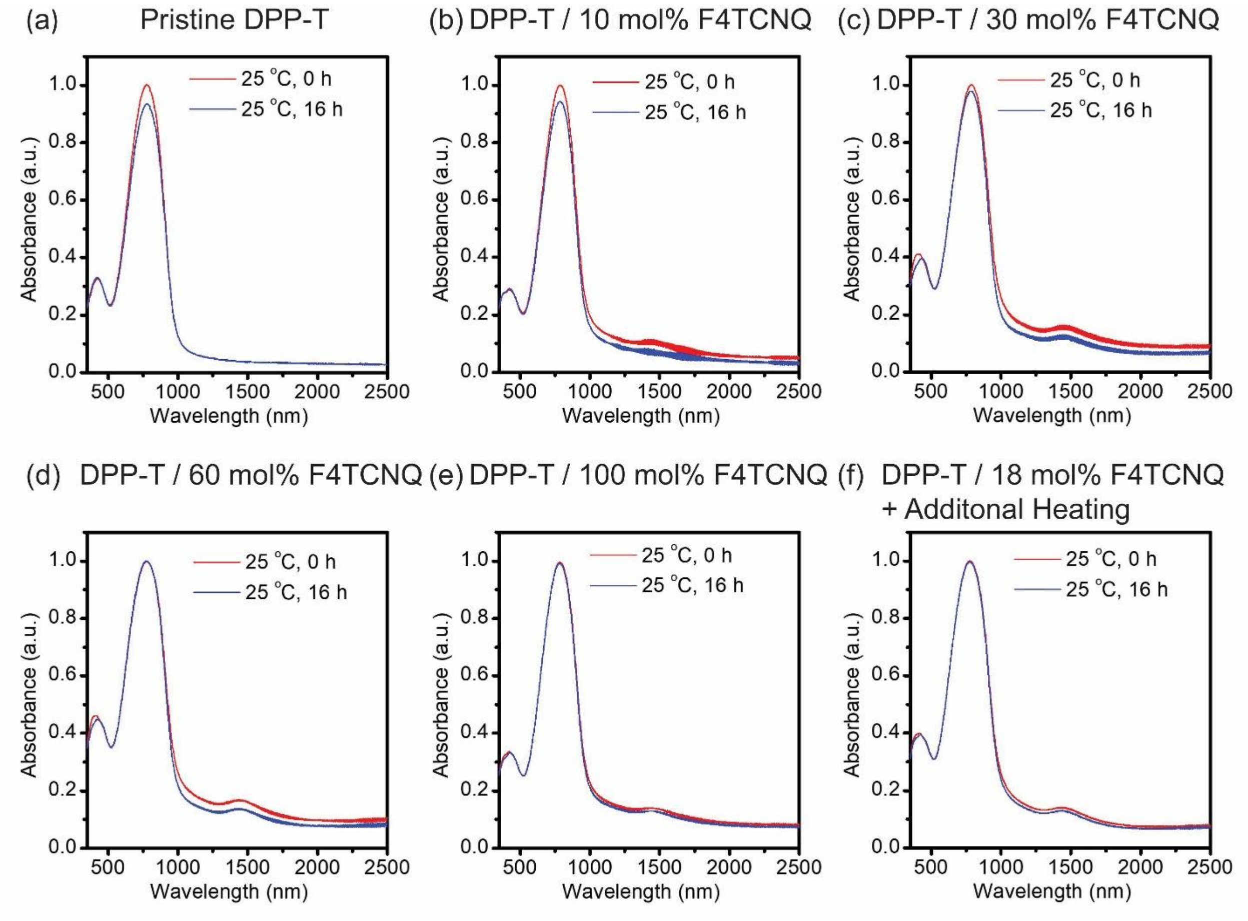

Figure S1. (a-e) UV-vis spectra of drop-casted films DPP-T doped with various doping ratios (10 mol% – 100 mol%) of F_4TCNQ before and after keeping at room temperature (25 °C) overnight (16 h). (f) UV-vis spectra (0h, 16h) of the drop-casted film DPP-T doped with 18 mol% F_4TCNQ with additional heating of the doping solution at 80 °C for 18h.

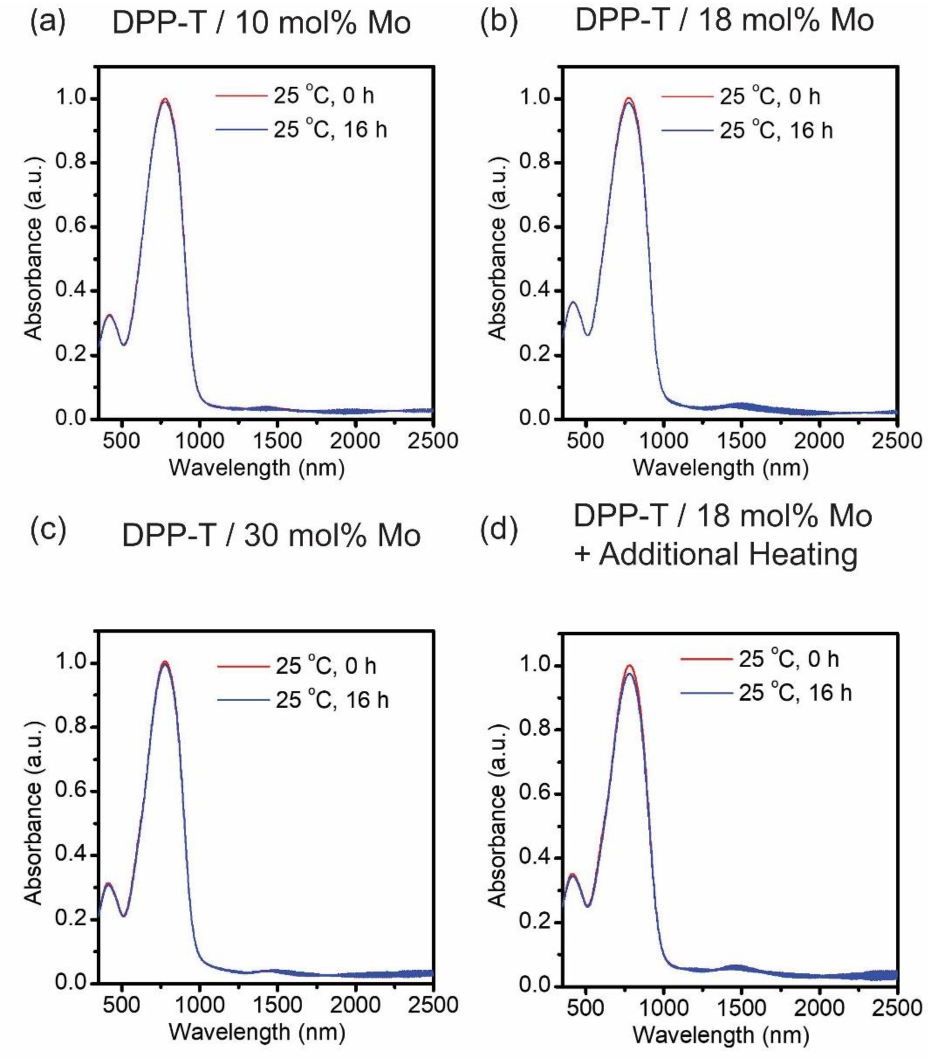

Figure S2. (a-c) UV-vis spectra of drop-casted films DPP-T doped with various doping ratios (10 mol% Mo – 30 mol% Mo) of Mo(tfd-CO_2Me)$_3$ before and after keeping at room temperature (25 °C) overnight (16 h). (d) UV-vis spectra (0h, 16h) of the drop-casted film DPP-T doped with 18 mol% Mo(tfd-CO_2Me)$_3$ with additional heating of the doping solution at 80 °C for 18h.

2. Processing parameters of spin-coated doped DPP-T-C_6C_8 films

Table S1. Processing parameters of each spin-coated doped film with its corresponding film thickness.

Entry	CP/dopant	CP concentration (mg/mL)	Dopant concentration (mg/mL)	Doping ratio	Spin rate (rpm)	Thickness[a] (nm)
1	DPP-T-C_6C_8/F_4TCNQ	15	0.2	10 mol%	500	~65
2	DPP-T-C_6C_8/F_4TCNQ	15	0.5	18 mol%	500	~73
3	DPP-T-C_6C_8/Mo(tfd-$CO_2Me)_3$	15	10	18 mol%	1200	~65
4	DPP-T-C_6C_8/Mo(tfd-$CO_2Me)_3$	15	10	18 mol%	700	~140

[a] Film thickness was determined by a profilometer.

3. Additional AFM images.

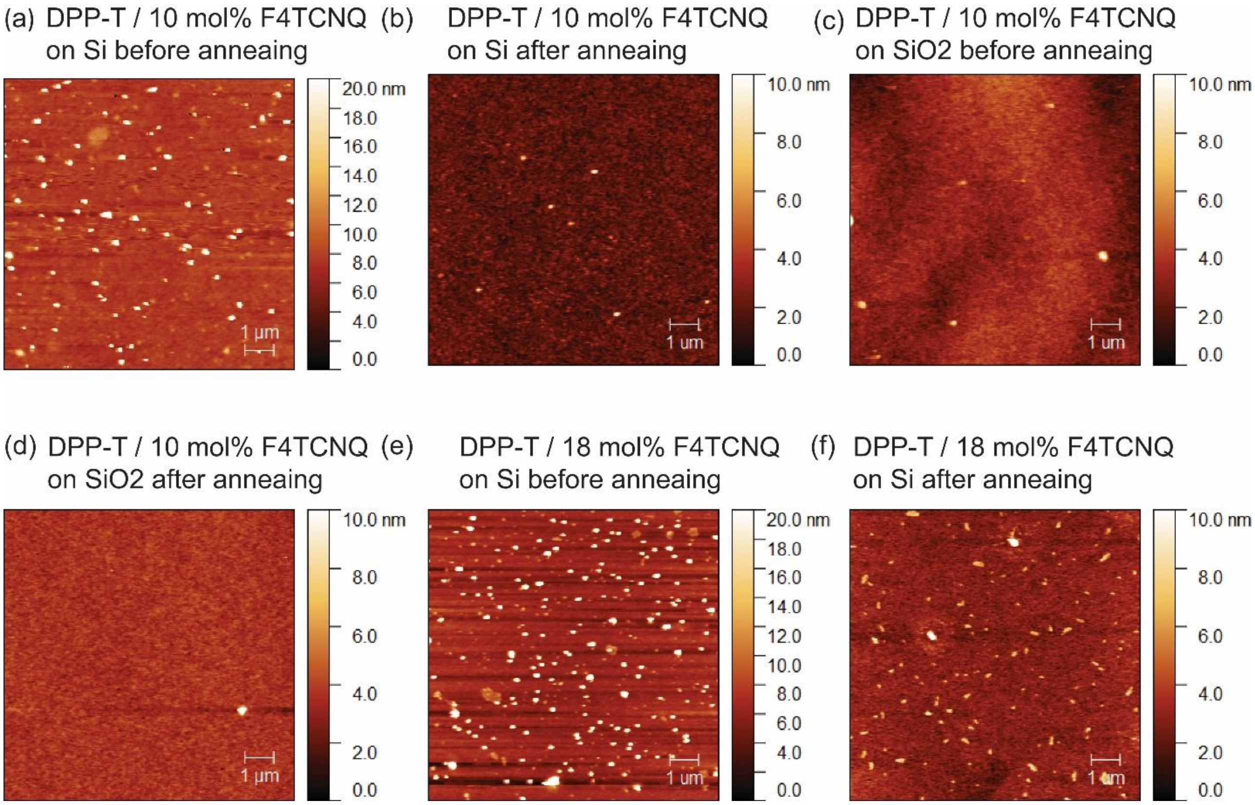

Figure S3. (a-f) AFM images of pristine and F_4TCNQ doped DPP-T films spin-coated from the doping solution mixtures on Si or SiO_2 substrates. (a) DPP-T doped with 10 mol% of F_4TCNQ before annealing on Si. (b) DPP-T doped with 10 mol% of F_4TCNQ after annealing on Si. (c) DPP-T doped with 10 mol% of F_4TCNQ before annealing on SiO_2. (d) DPP-T doped with 10 mol% of F_4TCNQ after annealing on SiO_2. (e) DPP-T doped with 18 mol% of F_4TCNQ before annealing on Si. (f) DPP-T doped with 18 mol% of F_4TCNQ after annealing on Si. The annealing was performed at 120 °C for 0.5 h. All images are in a size of 10 × 10 μm square.

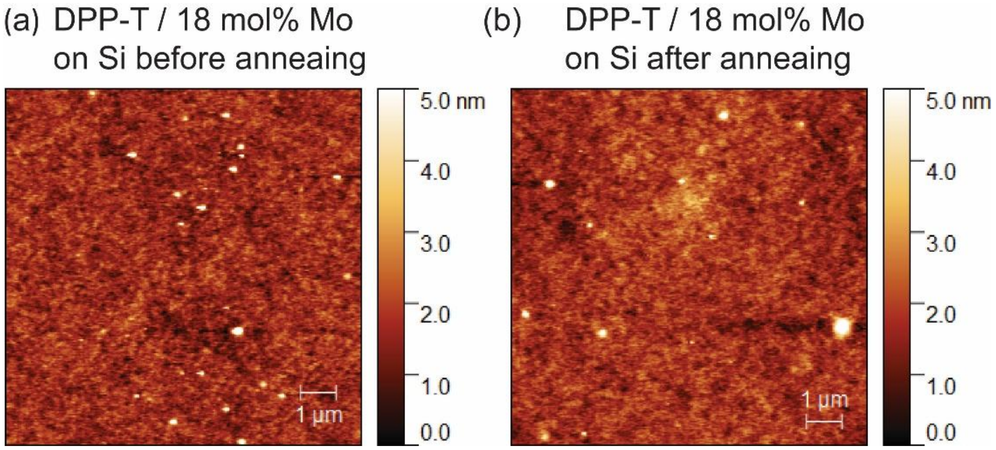

Figure S4. AFM images of pristine and Mo(tfd-CO_2Me)$_3$ doped DPP-T films spin-coated from the doping solution mixtures on Si or SiO_2 substrates. (a) DPP-T doped with 18 mol% of Mo(tfd-CO_2Me)$_3$ before annealing on Si. (b) DPP-T doped with 18 mol% of Mo(tfd-CO_2Me)$_3$ after annealing on Si. The annealing was performed at 120 °C for 0.5 h. All images are in a size of 10 × 10 μm square.

4. *I-V* curves of the doped DPP-T films under room temperature and after thermal annealing.

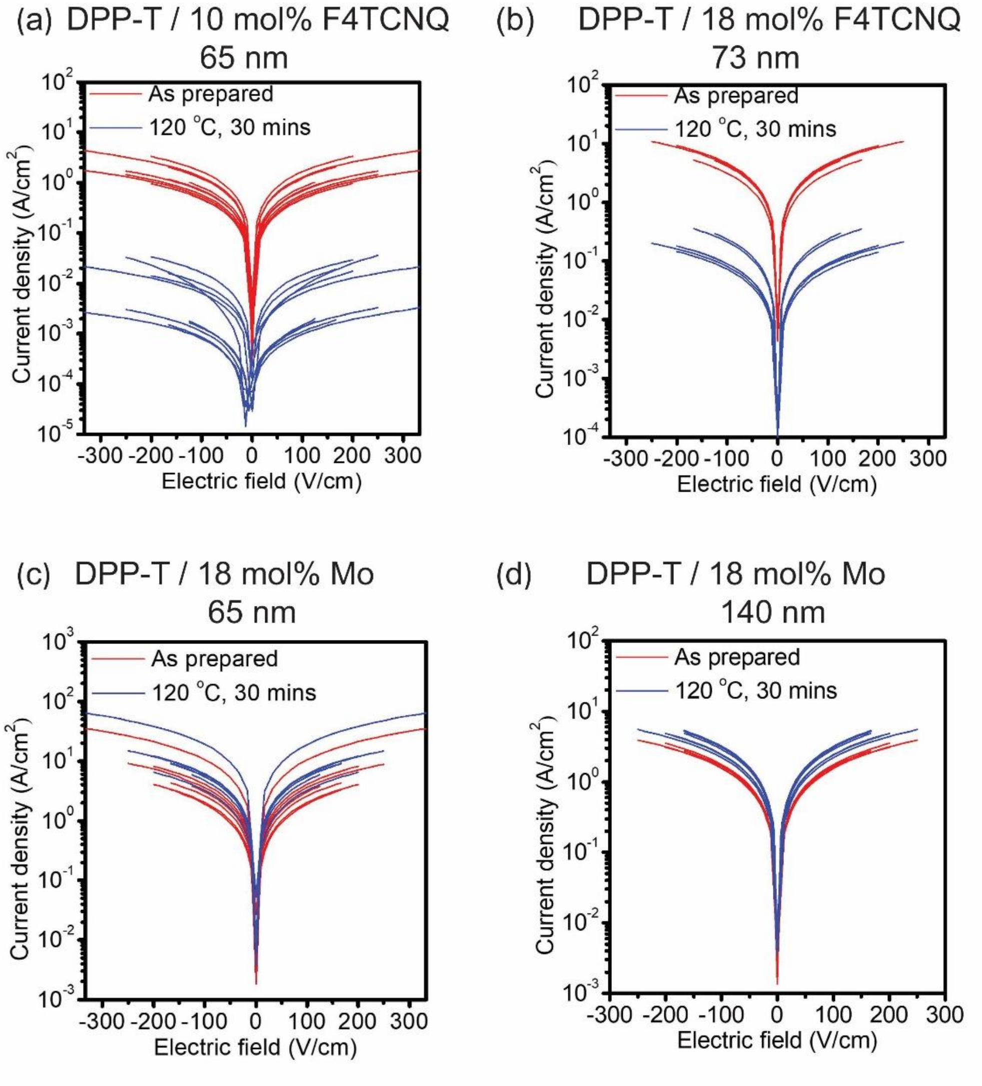

Figure S5. *I-V* curves of spun-coated doped DPP-T film with different thicknesses before and after thermal annealing at 120 °C for 30 minutes. Due to variable channel length (*L*) in the film devices, current (A) is normalized by film area ($W \times L$) into current density (A/cm^2), and voltage (V) is normalized by channel length (*L*) into electric field (V/cm).

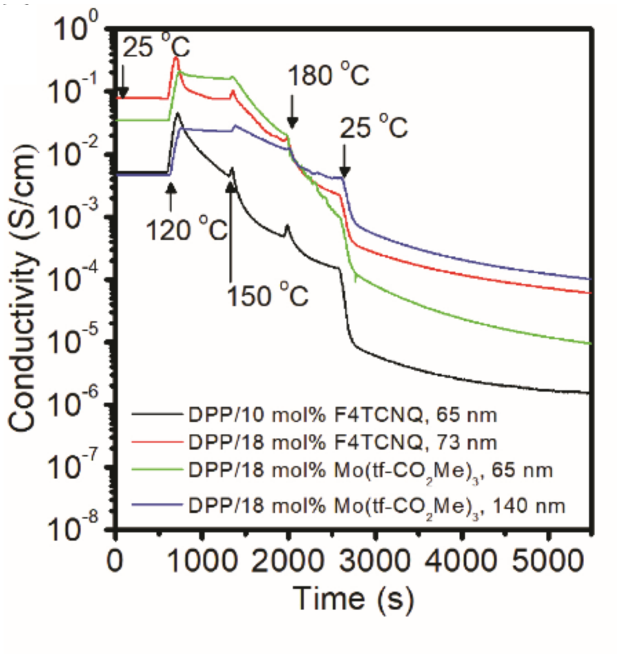

Figure S6. *I-V* curves of spun-coated doped DPP-T film with different thicknesses before and after thermal annealing at 120 °C. In-situ conductivity measurements of doped DPP-T films under a programmed temperature ramp from 25 °C to 200 °C, then cooled to room temperature. During heating, each temperature is held for 10 minutes.

5. Supplementary GIWAXS Results

Table S2. Summary of the Peak shifts, Alkyl Spacing and π Stacking Distance from GIWAXS analysis of the doped and neat DPP-T spin-coated films.

Peak Assignment	Neat, q (A^{-1})*	Mo-doped, q (A^{-1})	F_4TCNQ-doped, q (A^{-1})
(100)	0.35	0.35	0.33
(200)	0.65	0.65	0.65
(300)	N/A	N/A	N/A
(010)	1.68	N/A	N/A
Alkyl Spacing	17.94	17.94	19.03
π Stacking Distance	3.74	N/A	N/A

6. Supplementary Thermogravimetric analysis (TGA) Results

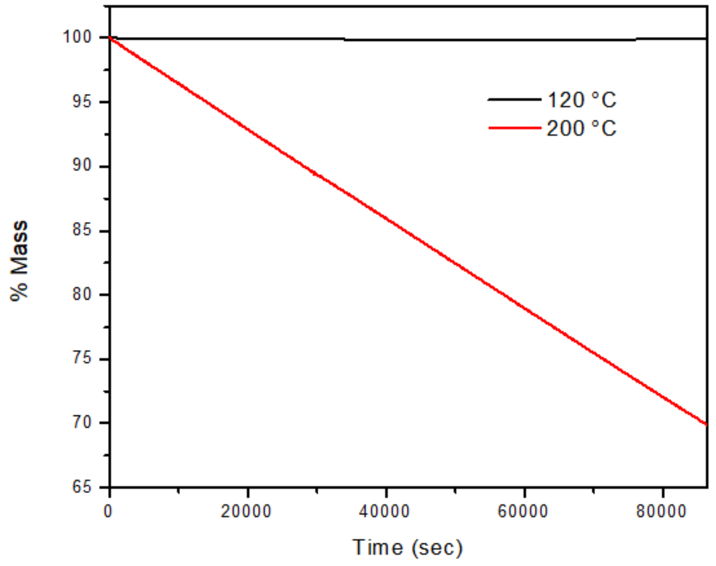

Figure S7. F_4TCNQ dopant isothermal TGA experiments at 120 °C and 200 °C for 24 h. There is no loss for the 120 °C, while the 200°C hold shows steady loss, with ~ 70% of the mass remaining after 24 h, indicating partial sublimation occurred.

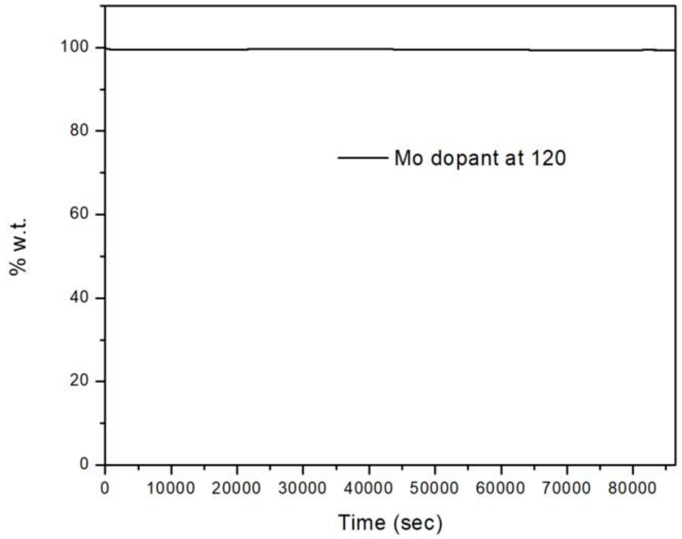

Figure S8. Mo dopant isothermal TGA experiment at 120°C for 24 h. There is no observed loss.

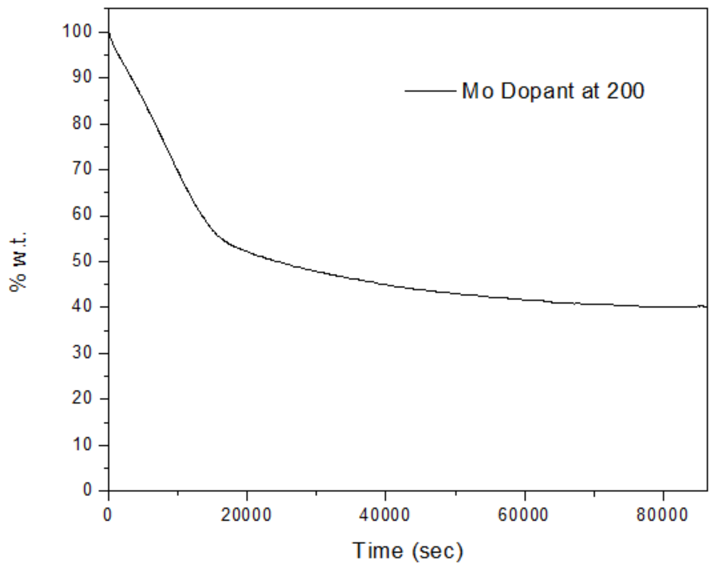

Figure S9. Mo dopant isothermal TGA experiment at 200 °C for 24 h. The mass loss was ~55% at the end of 24 h, indicating partial dopant sublimation.

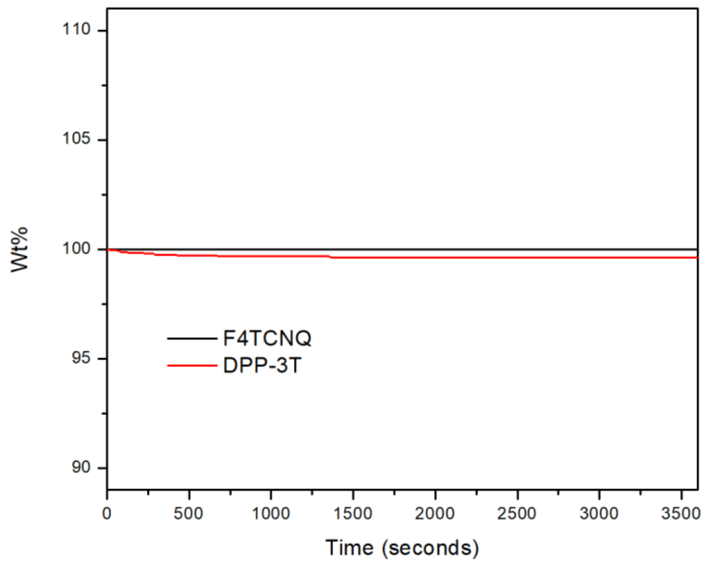

Figure S10. Isothermal TGA experiments of F_4TCNQ and DPP-3T polymer at 120 °C for 1h. No significant loss is observed in either sample. The small loss observed in the initial region of the DPP-3T is possibly due to the loss of chlorobenzene (CB) solvent residual.

7. Supplementary water contact angle analysis results of thin film DPP-T, F_4TCNQ, and $Mo(tfd\text{-}CO_2Me)_3$.

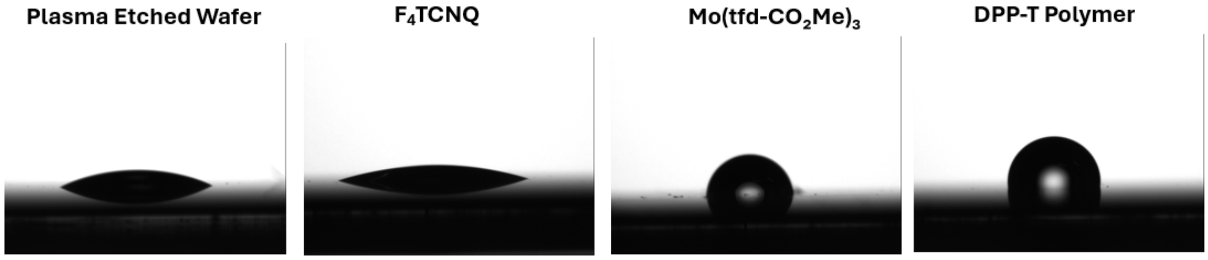

Plasma Etched wafer		F4TCNQ		Mo Dopant		DPP-T Polymer	
1	25.1	4	37.9	7	88.7	10	98.4
2	25.4	5	37.5	8	90.8	11	95.7
3	28.0	6	34.2	9	91.4	12	96.7
**avg**	**26.2**	avg	**36.5**	avg	**89.8**	avg	**96.9**
St.dev	1.6	St.dev	2.0	St.dev	1.4	St.dev	1.4

Figure S11. Contact angle analysis images of Plasma etched wafter, dopants, and polymer with table of results for the average water contact angle.

8. Supplementary FTIR spectra of bulk DPP-T, F_4TCNQ, and $Mo(tfd\text{-}CO_2Me)_3$ from AFM-IR

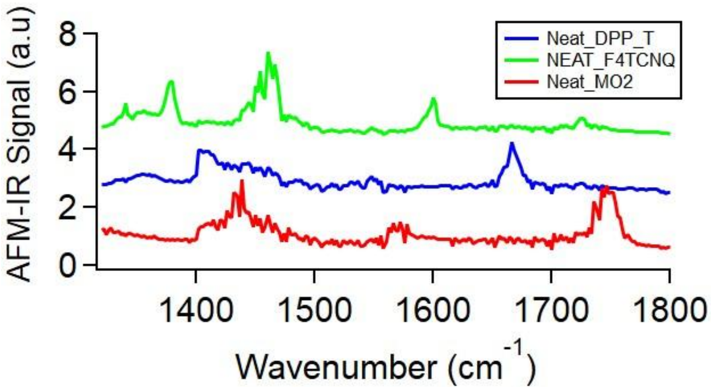

Figure S12. FTIR spectra of bulk DPP-T, F_4TCNQ, and Mo(tfd-CO2Me)$_3$. DPP-T film was prepared from spin-coating of a 15 mg/mL solution in chlorobenzene, while F_4TCNQ and Mo(tfd-CO2Me)$_3$ films were drop-casted from their solutions in chlorobenzene.

9. ATR-FTIR Spectra of DPP-T Polymer and Dopants

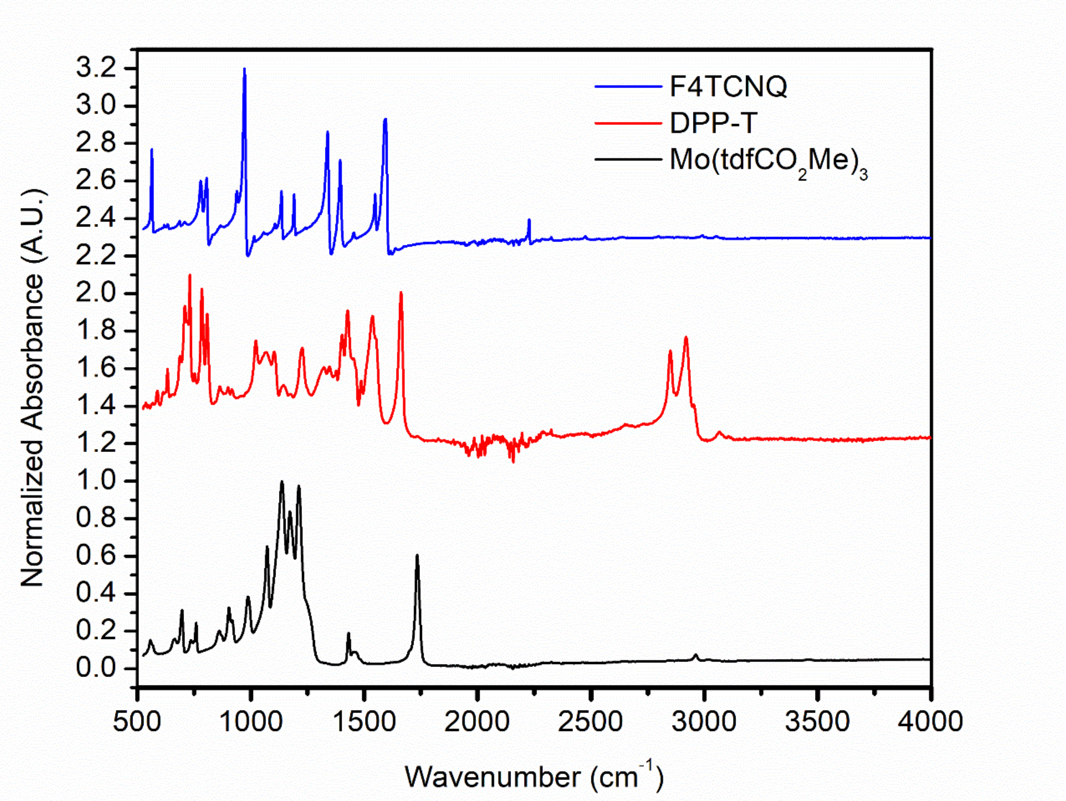

Figure S13. ATR-FTIR spectra of the dry powder form of the polymer (red), F_4TCNQ dopant (blue), and the Mo dopant (black).

10. UV-Vis study with sample preparation and heating performed in an inert glovebox.

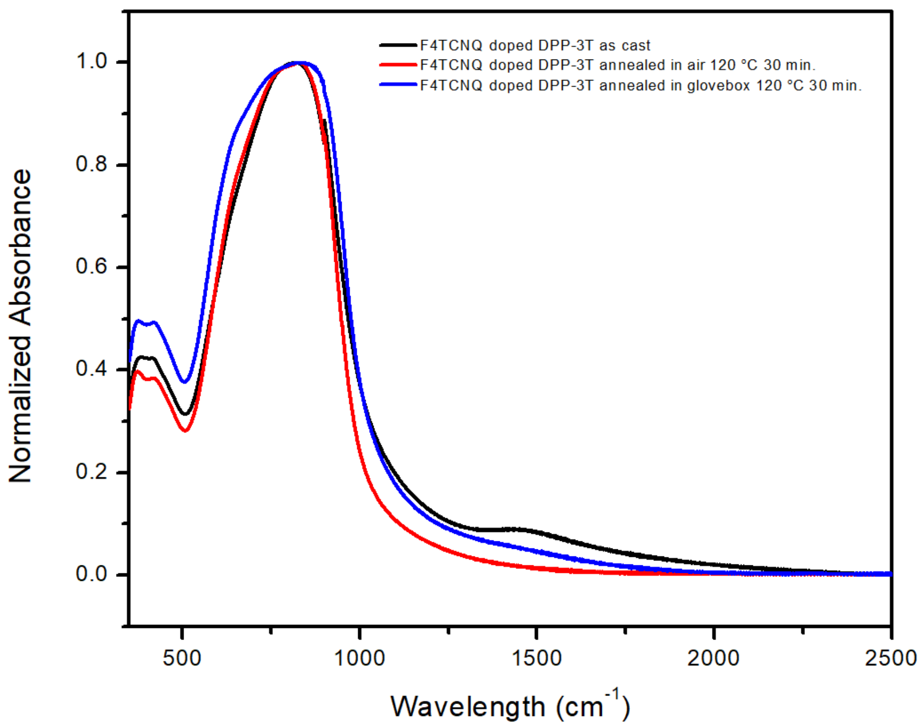

Figure S14.UV-vis spectra of F_4TCNQ doped sample as cast (black) in a glove box, heated (red) in air, and (blue) in the glove box. Scans were taken after the respective sample was heated at 120 °C for 30 minutes.

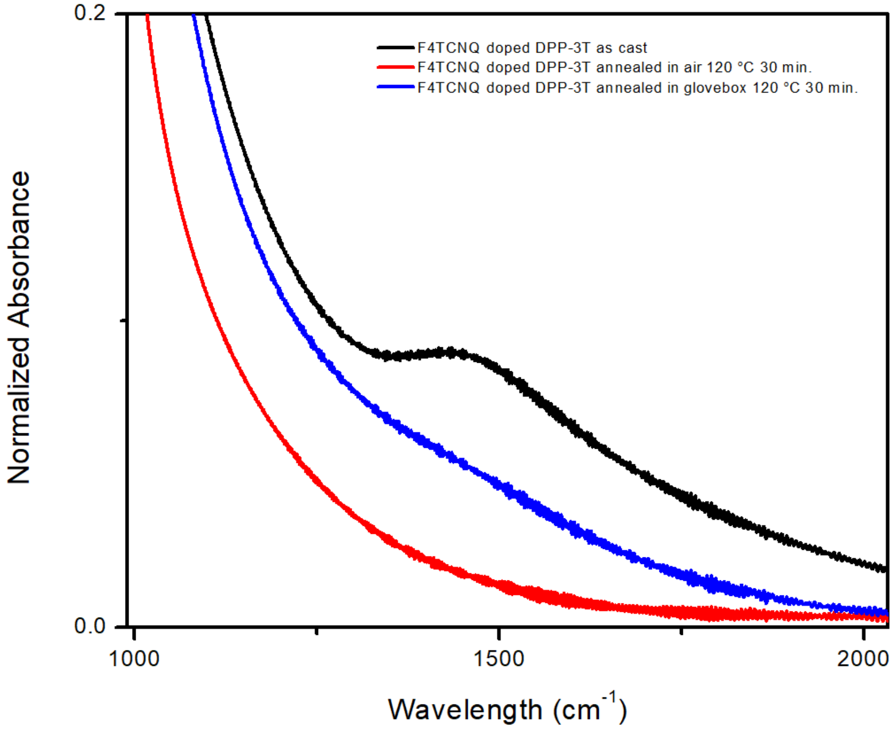

Figure S15.Zoomed in for polaronic band region UV-vis spectra of F_4TCNQ doped sample as cast (black) in a glove box, heated (red) in air, and (blue) in the glove box. Scans were taken after the respective sample was heated at 120 °C for 30 minutes.

11. Supplementary TOF-SIMS Point to Point normalization chemical depth profiling

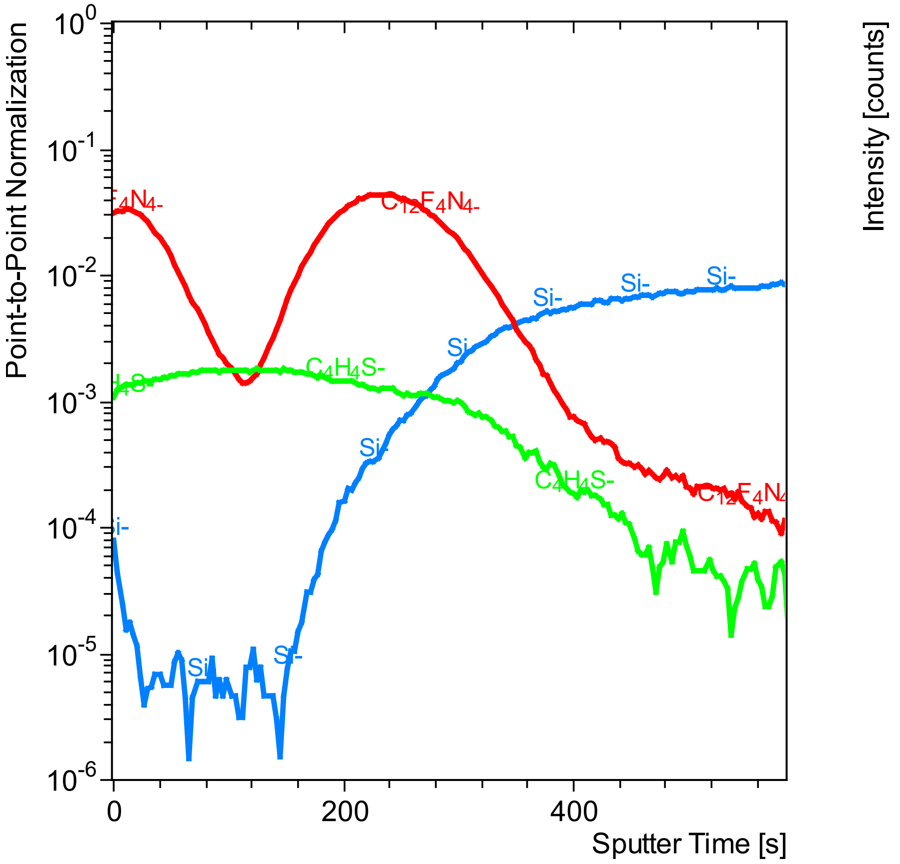

Figure S16. TOF-SIMS Point-to-Point normalization versus sputter time chemical depth profiling scan for F_4TCNQ doped as-cast sample spot 1.

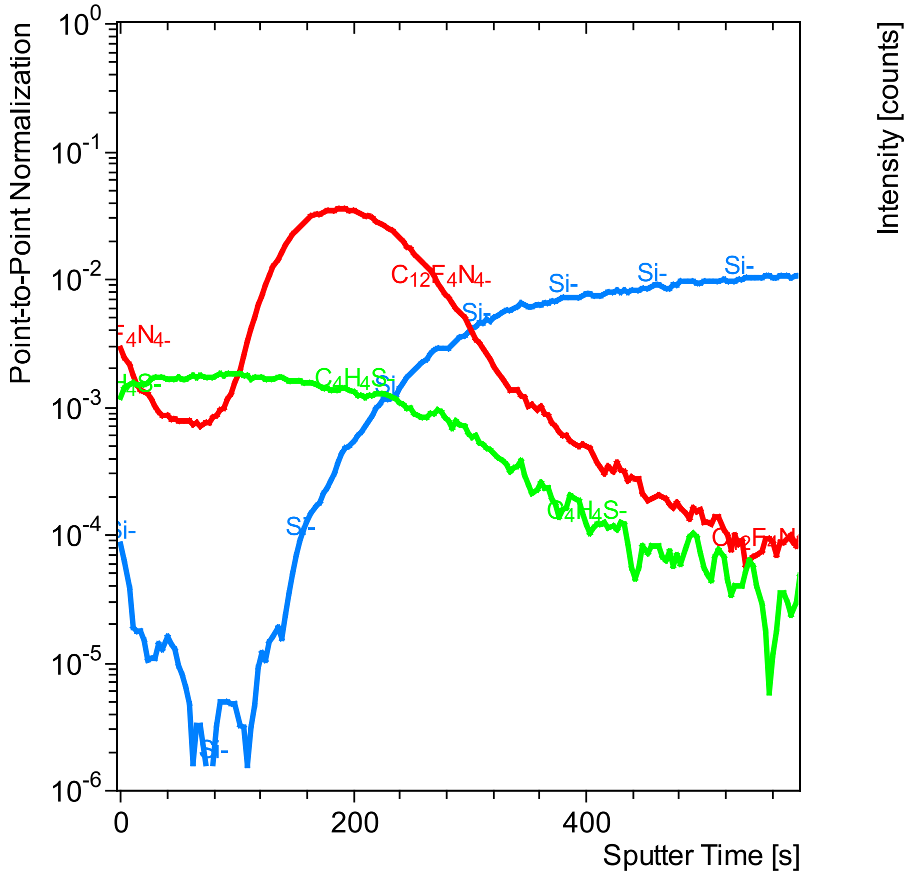

Figure S17. TOF-SIMS Point-to-Point normalization versus sputter time chemical depth profiling scan for F_4TCNQ doped as-cast sample spot 2

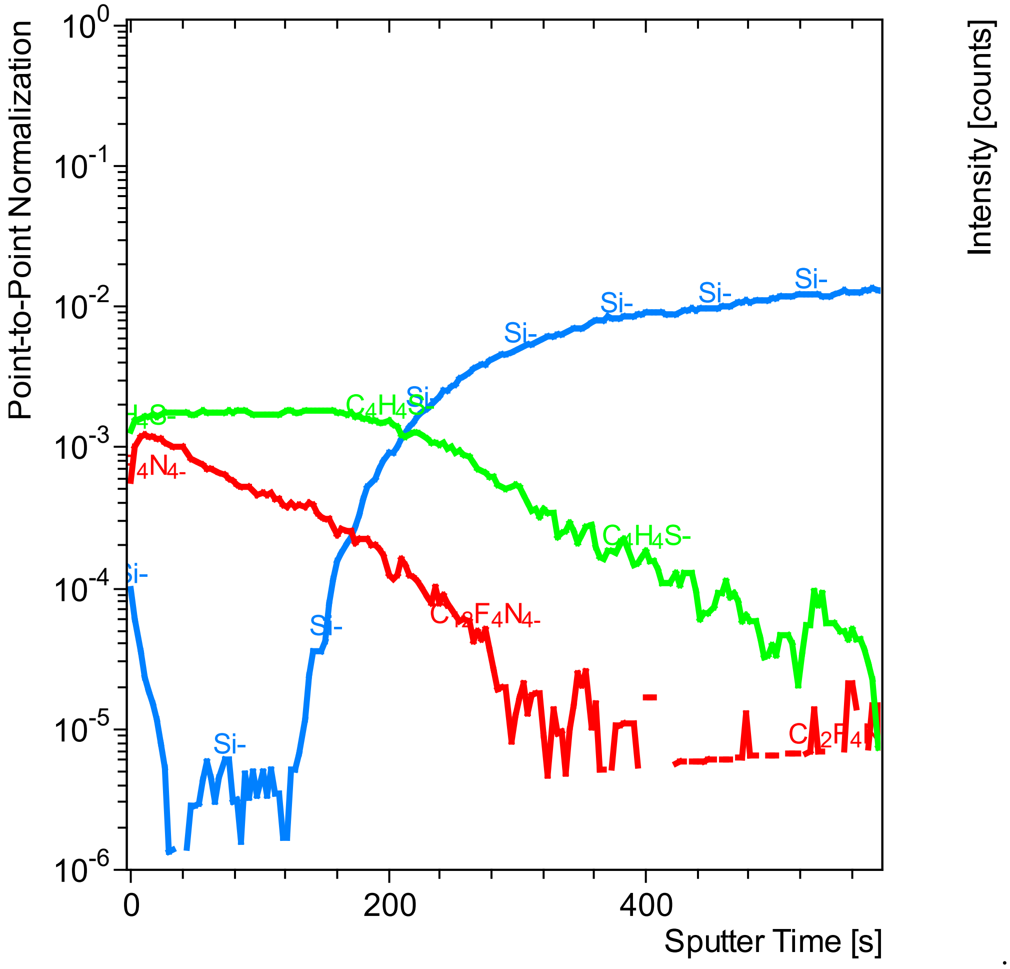

Figure S18. TOF-SIMS Point-to-Point normalization versus sputter time chemical depth profiling scan for F_4TCNQ doped annealed sample spot 1.

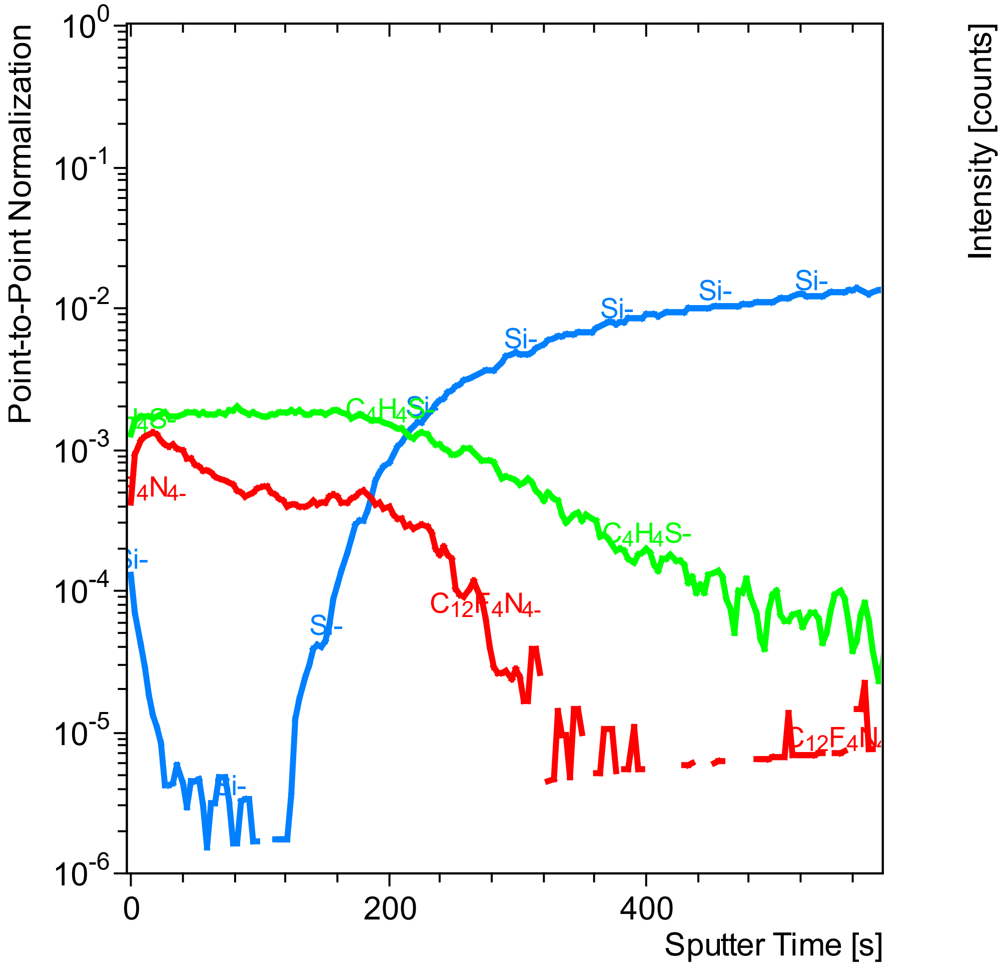

Figure S19. TOF-SIMS Point-to-Point normalization versus sputter time chemical depth profiling scan for F_4TCNQ doped annealed sample spot 2.

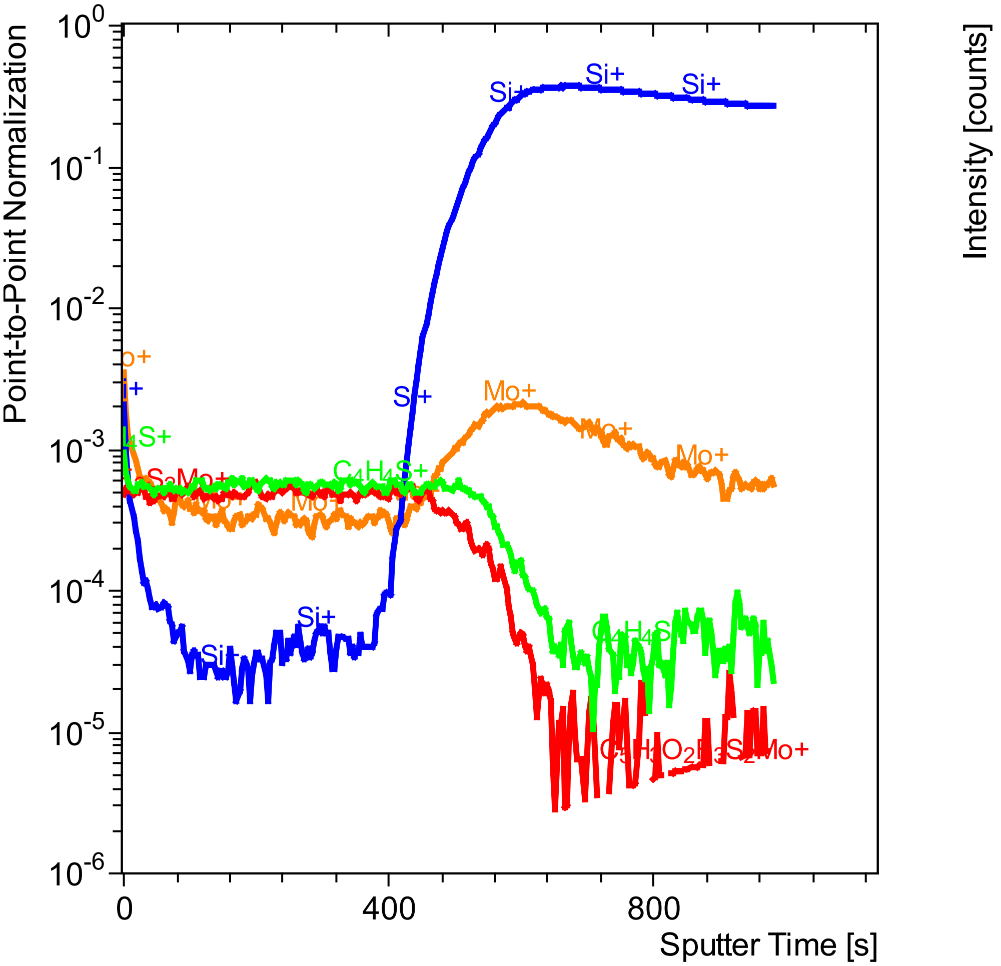

Figure S20. TOF-SIMS Point-to-Point normalization versus sputter time chemical depth profiling scan for Mo(tfd-CO_2Me)$_3$ doped as-cast sample spot 1.

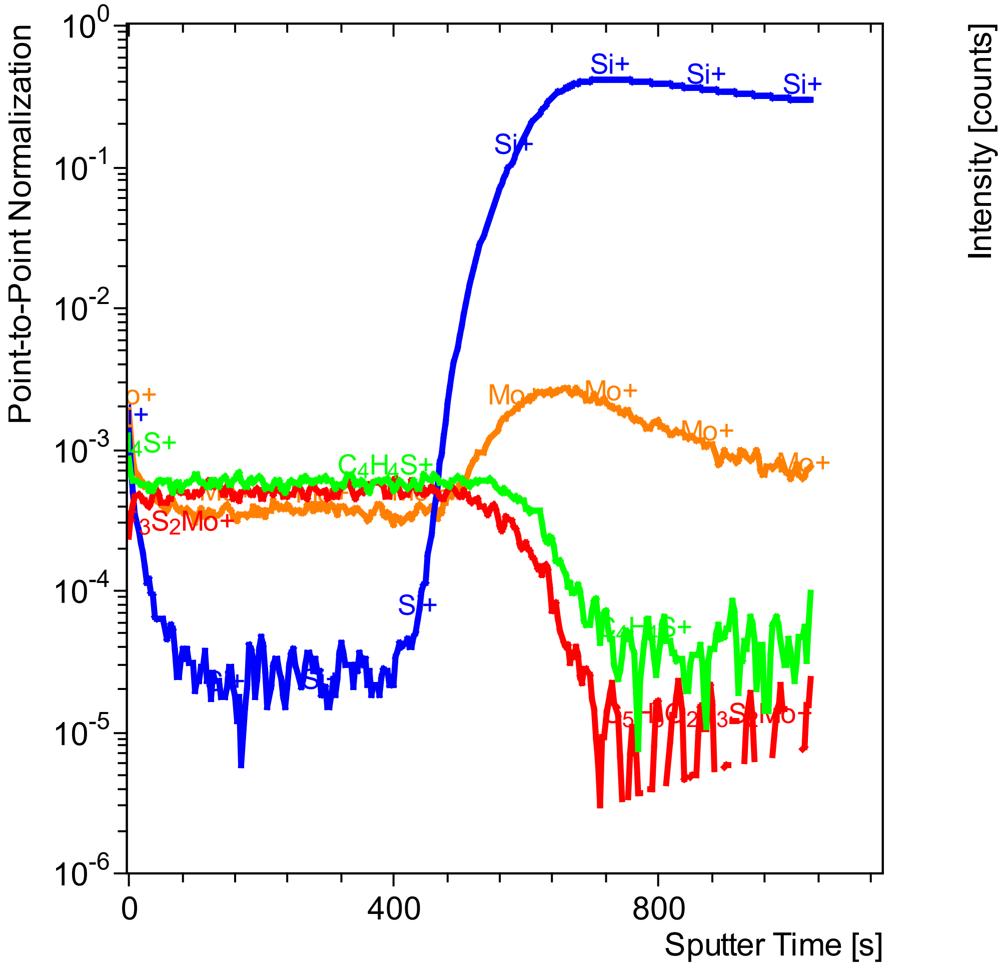

Figure S21. TOF-SIMS Point-to-Point normalization versus sputter time chemical depth profiling scan for Mo(tfd-CO_2Me)$_3$ doped as-cast sample spot 2.

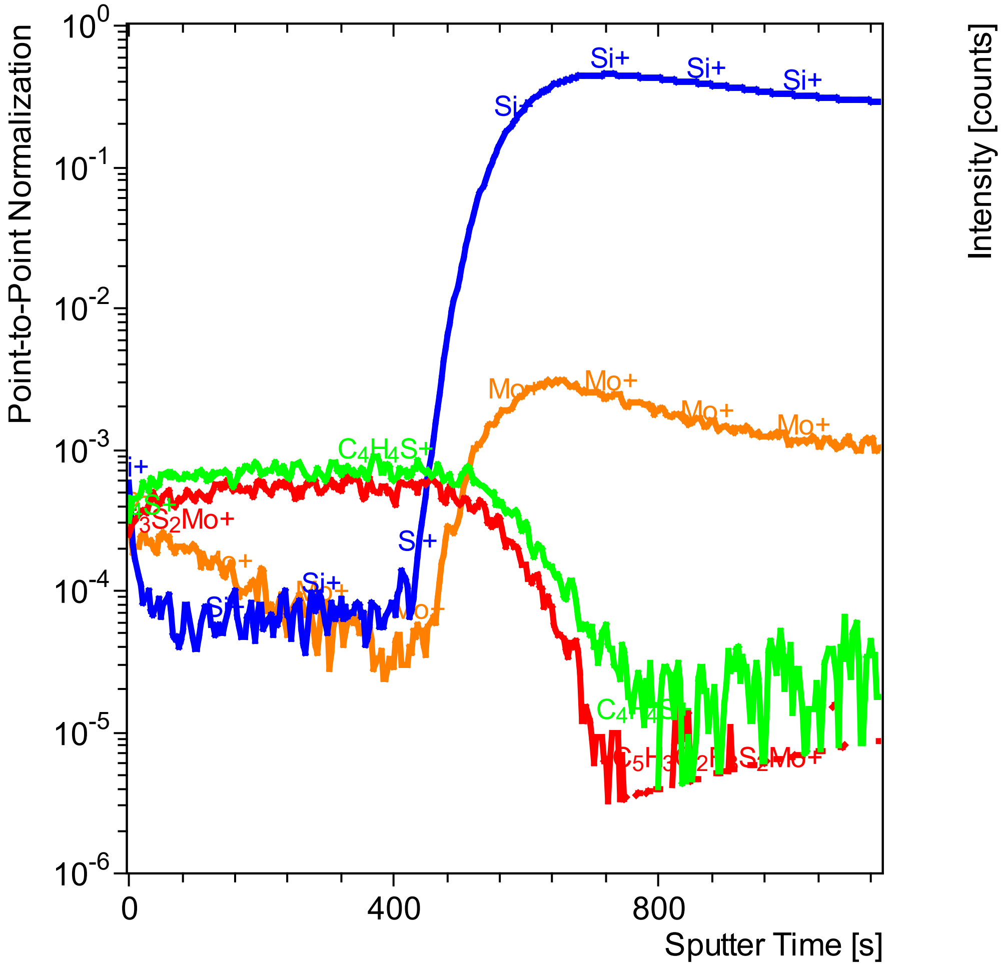

Figure S22. TOF-SIMS Point-to-Point normalization versus sputter time chemical depth profiling scan for Mo(tfd-CO_2Me)$_3$ doped annealed sample spot 1.

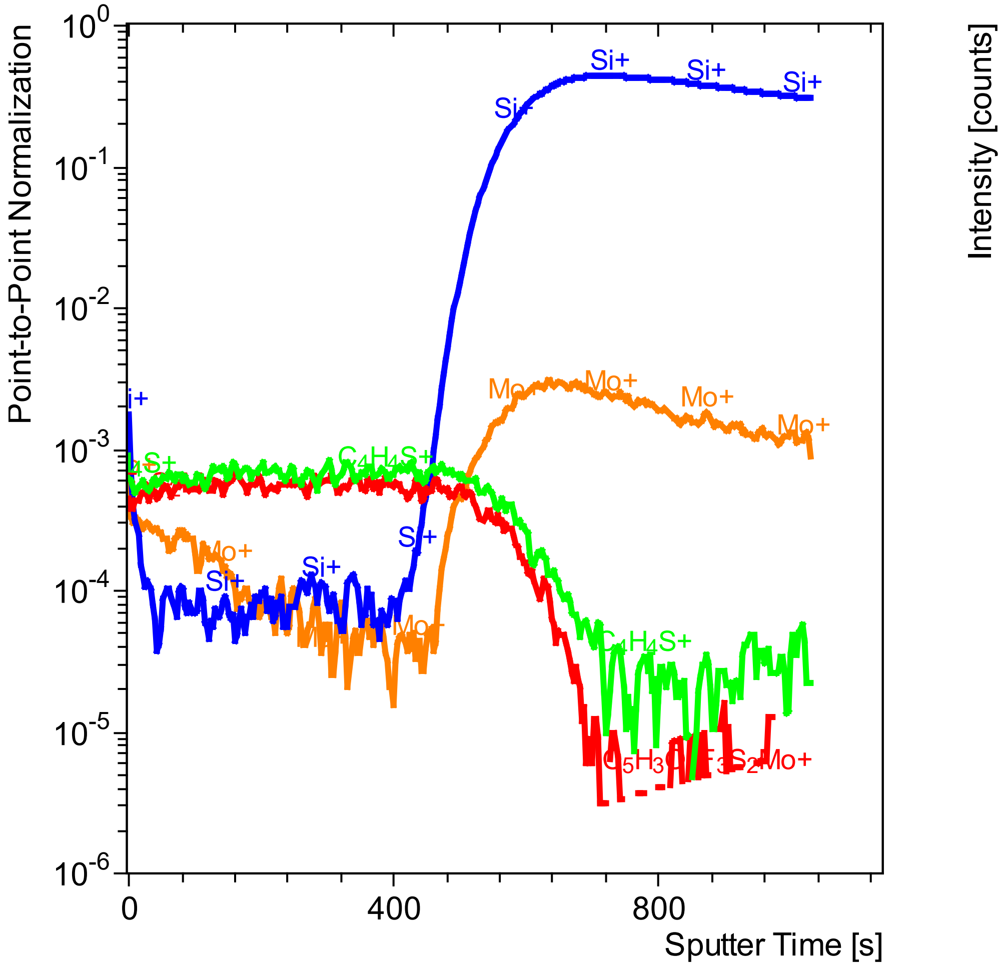

Figure S23. TOF-SIMS Point-to-Point normalization versus sputter time chemical depth profiling scan for Mo(tfd-$CO_2Me)_3$ doped annealed sample spot 2.
